# An Empirical Bayes Mixture Model for Effect Size Distributions in Genome-Wide Association Studies

**DOI:** 10.1371/journal.pgen.1005717

**Published:** 2015-12-29

**Authors:** Wesley K. Thompson, Yunpeng Wang, Andrew J. Schork, Aree Witoelar, Verena Zuber, Shujing Xu, Thomas Werge, Dominic Holland, Schizophrenia Working Group of the Psychiatric Genomics Consortium, Ole A. Andreassen, Anders M. Dale

**Affiliations:** 1 Institute of Biological Psychiatry, Mental Health Centre Sct. Hans, Mental Health Services, Copenhagen, Denmark; 2 The Lundbeck Foundation Initiative for Integrative Psychiatric Research, iPSYCH, Copenhagen, Denmark; 3 Department of Psychiatry, University of California, San Diego, La Jolla, California, United States of America; 4 Division of Mental Health and Addiction, Oslo University Hospital, Oslo, Norway; 5 Department of Cognitive Science, University of California, San Diego, La Jolla, California, United States of America; 6 Department of Clinical Medicine, University of Copenhagen, Copenhagen, Denmark; 7 Multimodal Imaging Laboratory, University of California at San Diego, La Jolla, California, United States of America; Baylor College of Medicine, UNITED STATES

## Abstract

Characterizing the distribution of effects from genome-wide genotyping data is crucial for understanding important aspects of the genetic architecture of complex traits, such as number or proportion of non-null loci, average proportion of phenotypic variance explained per non-null effect, power for discovery, and polygenic risk prediction. To this end, previous work has used effect-size models based on various distributions, including the normal and normal mixture distributions, among others. In this paper we propose a scale mixture of two normals model for effect size distributions of genome-wide association study (GWAS) test statistics. Test statistics corresponding to null associations are modeled as random draws from a normal distribution with zero mean; test statistics corresponding to non-null associations are also modeled as normal with zero mean, but with larger variance. The model is fit via minimizing discrepancies between the parametric mixture model and resampling-based nonparametric estimates of replication effect sizes and variances. We describe in detail the implications of this model for estimation of the non-null proportion, the probability of replication in *de novo* samples, the local false discovery rate, and power for discovery of a specified proportion of phenotypic variance explained from additive effects of loci surpassing a given significance threshold. We also examine the crucial issue of the impact of linkage disequilibrium (LD) on effect sizes and parameter estimates, both analytically and in simulations. We apply this approach to meta-analysis test statistics from two large GWAS, one for Crohn’s disease (CD) and the other for schizophrenia (SZ). A scale mixture of two normals distribution provides an excellent fit to the SZ nonparametric replication effect size estimates. While capturing the general behavior of the data, this mixture model underestimates the tails of the CD effect size distribution. We discuss the implications of pervasive small but replicating effects in CD and SZ on genomic control and power. Finally, we conclude that, despite having very similar estimates of variance explained by genotyped SNPs, CD and SZ have a broadly dissimilar genetic architecture, due to differing mean effect size and proportion of non-null loci.

## Author Summary

We describe in detail the implications of a particular mixture model (a scale mixture of two normals) for effect size distributions from genome-wide genotyping data. Parameters from this model can be used for estimation of the non-null proportion, the probability of replication in *de novo* samples, the local false discovery rate, power for detecting non-null loci, and proportion of variance explained from additive effects. Here, we fit this model by minimizing discrepancies with nonparametric estimates from a resampling-based algorithm. We examine the effects of linkage disequilibrium (LD) on effect sizes and parameter estimates, both analytically and in simulations. We validate this approach using meta-analysis test statistics (“z-scores”) from two large GWAS, one for Crohn’s disease and the other for schizophrenia. We demonstrate that for these studies a scale mixture of two normal distributions generally fits empirical replication effect sizes well, providing an excellent fit for the schizophrenia effect sizes but underestimating the tails of the distribution for Crohn’s disease.

## Introduction

While genome-wide association studies (GWAS) have discovered thousands of genome-wide significant risk loci for heritable disorders, including Crohn’s disease [1] and schizophrenia [2], so far even large meta-analyses have recovered only a fraction of the heritability of most complex traits. Some of this “missing heritability” may be due to rare variants of large effect, epistasis, copy-number variation, epigenetics, etc. However, recent work utilizing variance components models [2–5] has demonstrated that a much larger fraction of the heritability of complex phenotypes is captured by the additive effects of SNPs than is evident only in loci surpassing genome-wide significance thresholds. Thus, the emerging picture is that traits such as these are highly polygenic, and that the heritability is largely accounted for by numerous loci each with a very small effect [5, 6]. In this scenario, instead of estimating effect sizes individually, it is useful to characterize the *distribution* of effect sizes for choosing significance thresholds, for estimation of power, for the computation of an individual’s overall genetic risk for a disease, and for the identification of disease mechanisms that can be used for the development of effective treatments.

Effect size distributions can be estimated directly from the genotype-phenotype data [3, 7–10] or from the summary statistics produced from GWAS analyses [11, 12]. In this paper we focus on estimation of effect size distributions from summary statistics, produced from fitting a regression model for each single nucleotide polymorphism (SNP) individually. A Wald test statistic (“*z*-score”) is computed from the regression of each SNP to test its association with the phenotype of interest. A SNP is often declared significant if the *p*-value of its test statistic surpasses a Bonferroni-inspired threshold of 5 × 10^−8^. Note, within this typical GWAS hypothesis testing framework, the effect size for a given SNP computed from massively univariate test statistics is a weighted combination of effects from all SNPs that it is in linkage disequilibrium (LD) with (see [13] as well as S1 Text for more details).

An implicit assumption in GWAS hypothesis testing is that SNP test statistics come from a mixture distribution of zero (null) and non-zero (non-null) effect sizes [14], though this mixture distribution is not usually explicitly modeled. The values of parameters from such a mixture distribution characterize important aspects of the genetic architecture of a phenotype, including the proportion of non-null effects, the variance explained per non-null locus, and the amount of inflation in the null distribution [15]. Mixture model parameters can also be used to compute other quantities of interest, including estimates of the probability of replication in a *de novo* study, the posterior probability that a given SNP is null or has a negligible effect conditional on its observed *z*-score (i.e., the local false discovery rate), and the power to detect susceptibility loci for a given study sample size. These parameters are also closely related to the proportion of the phenotypic variance explained by the additive effects of common variants and upper limits on the accuracy of polygenic risk scores [12, 16]. Information such as LD or the functional role of SNPs can be incorporated into the model to provide characterizations of the genetic architecture of complex disorders that do not implicitly assume that all SNPs are *a priori* exchangeable [17, 18].

In this paper we implement a simple scale mixture of two normals distribution to model GWAS *z*-scores. Test statistics corresponding to “null” associations are modeled as random draws from a normal distribution with zero mean; test statistics corresponding to “non-null” associations are also modeled as random draws from a normal distribution with zero mean but with larger variance. The proportion of tests corresponding to null associations is also estimated. (This model has a Bayesian interpretation, and the methods proposed are “empirical Bayes” because the prior probability of being null is estimated from the data [19].) A closely related model has been previously proposed for GWAS effect sizes using genotype-phenotype data [10].

We derive the connection between this mixture model and the finite-sample probability of replication in *de novo* samples, the local false discovery rate, and the power for detecting a specified proportion of the phenotypic variance due to additive effects of genetic loci for a given local false discovery rate. The mixture model is fitted using a resampling-based procedure applied to meta-analysis sub-study *z*-scores. By repeatedly and randomly partitioning the sub-studies into disjoint training and replication samples, we obtain nonparametric smoothed estimates of replication effect sizes and variances that are scaled estimates of their conditional posterior expectations (given the observed z-scores) with respect to a simple measurement model. We then fit a parametric scale mixture of two normals models that minimizes the sum of squared discrepancies with these nonparametric estimates.

We demonstrate this statistical framework in simulations and on meta-analysis *z*-scores from Crohn’s disease [1] and schizophrenia [20] GWAS. We show that the scale mixture of two normals model provides an excellent fit to the posterior effect size means and variances for the schizophrenia data, while capturing the general behavior (though underestimating the tails of the effect size distribution) for Crohn’s disease. We conclude that, despite having very similar estimates of variance explained by genotyped SNPs, Crohn’s disease and schizophrenia have a broadly dissimilar genetic architecture due to differing mean effect size and proportion of non-null loci. Finally, we examine the effects of LD on effect size distributions estimated from GWAS summary statistics, both analytically and in simulation studies.

## Results

### Crohn’s Disease

Crohn’s disease (CD) is a type of inflammatory bowel disease that is caused by multiple factors in genetically susceptible individuals. Estimates of narrow-sense heritability for CD are *h*^2^ ≈ 0.50 [21]. The variance captured by the additive effects of genotyped SNPs using a liability model assuming an underlying normal distribution for additive per allele risk effects has been estimated at $h_{\text{chip}}^{2}=0.22$ [22]. The CD data consist of *N* = 942,772 SNP *z*-scores from a GWAS meta-analysis of eight sub-studies on a total of *n* = 23,671 subjects (7,352 cases) [1]. Sub-study *z*-scores are available at http://www.ibdgenetics.org/downloads.html. Before running the resampling algorithm, SNPs were randomly pruned for approximate independence, so that LD ≤ 0.20 between any pair of SNPs, resulting in *N* = 97,855 SNPs.

**Fig 1:**
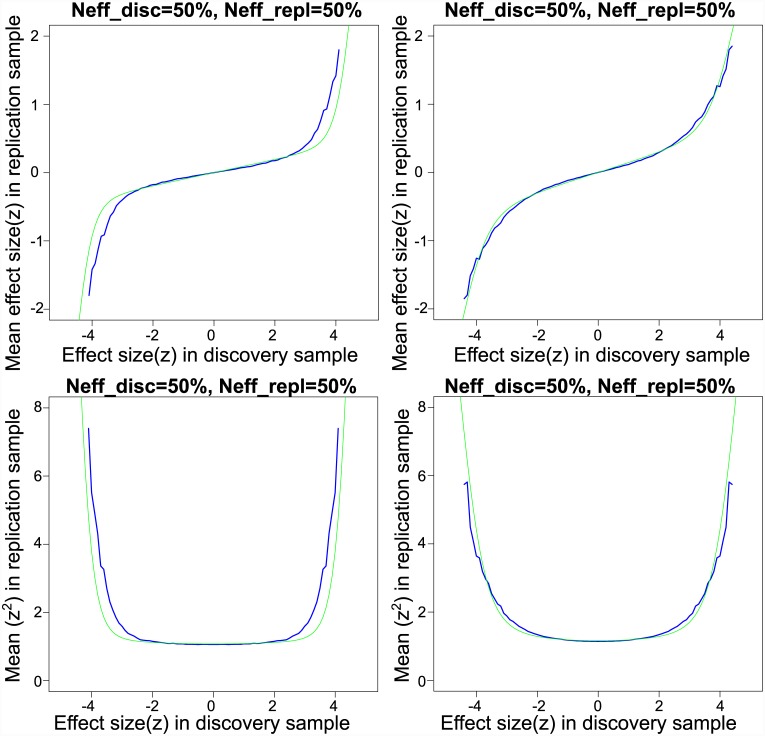
Empirical and model-based posterior expectations and variances for schizophrenia and Crohn’s disease. *Upper left panel*: Schizophrenia empirical conditional mean of split-half replication *z*-scores (purple line) and fitted effect sizes from scale mixture of normals model (yellow line). *Lower left panel*: Schizophrenia empirical conditional variance of split-half replication *z*-scores (purple line) and fitted variances from scale mixture of normals model (yellow line). *Upper right panel*: Crohn’s disease empirical conditional mean of split-half replication *z*-scores (purple line) and fitted effect sizes from scale mixture of normals model (yellow line). *Lower right panel*: Crohn’s disease empirical conditional variance of split-half replication *z*-scores (purple line) and fitted variances from scale mixture of normals model (yellow line).

Fig 1 shows the resampling means and variances of replication *z*-scores as a function of training *z*-scores for the CD meta-analysis sub-studies, based on all 70 possible partitions of sub-studies into four training and four replication datasets. Also plotted are the predicted replication conditional means and variances from the best fitting scale mixture of two normals model. The nonparametric and model-based estimates show good agreement except in the tails (absolute discovery *z*-scores > 3). Lack of fit is due to larger effect sizes in the tails than is predicted by the mixture model. Stated differently, the distribution of effect sizes has a larger kurtosis than can be captured by the two-component mixture. This results in conservative estimates of replication effect sizes, replication probabilities, and local fdr for SNPs in this part of the distribution. Other authors have proposed a scale mixture including more than two components (e.g., [10]), which could be implemented within our resampling-based algorithm at the cost of two parameters per additional mixture component.

The estimated non-null proportion is ${\hat{\pi }}_{2}=0.0008$ indicating that almost 0.1% of the 97,855 approximately independent SNPs fall within in the “large effects” category. The standard deviation for small effects is ${\hat{\sigma }}_{1}=0.008$, and the standard deviation for large effects is ${\hat{\sigma }}_{2}=0.078$. The estimated null standard deviation is ${\hat{\sigma }}_{0}=0.991$, or slightly below the theoretical null standard deviation. Note, the “empirical null” variance [23] is approximately given by ${\hat{\sigma }}_{0}^{2}+2\bar{p}\left(1-\bar{p}\right)n{\hat{\sigma }}_{1}^{2}=1.08$, where *n* is the effective sample size of the study and $\bar{p}$ is the mean minor allele frequency. As indicated by the small but non-zero estimate of *σ*_1_, there is a positive slope through the origin in the plot of replication effects (upper left panel of Fig 1), indicating that even very small *z*-scores tend to replicate at a higher rate than expected by chance. Thus, it is more appropriate to state that replication *z*-scores show a mixture of “small” and “large” replicating effects rather than “null” and “non-null”. Small replicating effects could potentially be due to population stratification or to weak yet pervasive LD with causal effects (see S1 Text).

The estimated number of large effect SNPs among the 97,855 is given by $N\hat{\pi_{2}}=76$. There are 45 SNPs declared significant using a local fdr threshold of 0.05, which corresponds to SNPs with *p*-values ≤ 9.8 × 10^−8^. Thus, the CD meta-analysis is currently powered to detect approximately 60% of large effect SNPs using a local fdr threshold of 0.05.

Note, the presence of correlation among genetic loci due to LD is important for the interpretation of parameters in the mixture model. For example, the proportion of large effects *π*_2_ is dependent on the level of pruning, with *π*_2_ being larger in unpruned data and lower in data pruned for approximate independence. This is because large effects tend to be in higher total LD with other SNPs, and hence a higher proportion of these are eliminated during random pruning. One explanation why large-effect SNPs tend to have higher total LD is that these SNPs tag larger genomic regions and hence have a higher probability of tagging causal effects (see [13] and the S1 Text). Another possible explanation, not mutually exclusive with the first, is that SNPs that fall in functional genomic categories (e.g., within genes) are enriched for causal effects and that these categories also tend to be in regions of higher total LD [17, 18]. The balance between these two explanations determines how much *π*_2_ is over-estimated using unpruned loci or under-estimated using loci pruned for independence, relative to the underlying and unknown proportion of causal effects. While we perform random pruning to approximate independence here, the efficient and accurate handling of the effects of LD-induced correlation and blurring of effect size distributions is an area of on-going research.

### Schizophrenia

Schizophrenia (SZ) is known to be highly polygenic and has an estimated narrow-sense heritability *h*^2^ ≈ 0.8 [24]. The additive variance captured by SNPs using a liability model has been estimated at $h_{\text{chip}}^{2}=0.23$ [25], close to that of CD. The SZ data analyzed here consist of *N* = 2,558,411 association *z*-scores from a GWAS meta-analysis of 52 sub-studies with *n* = 82,315 total subjects (35,476 cases) [26]. The full study meta-analysis statistics are available at http://www.med.unc.edu/pgc/downloads. PGC analytic datasets can be obtained by application to the controlled-access NIMH Genetics Repository. Data were randomly pruned for pairwise LD ≤ 0.20, leaving *N* = 129,973 roughly independent SNPs. The resampling procedure was run over 100 iterations, with random splits of the sub-studies into differing proportions (30%,40%, and 50%) for training and the remaining proportion as replication data.

Fig 1 shows the empirical replication means and variances of *z*-scores, as a function of training *z*-scores, for the SZ meta-analysis sub-studies, based on the split-half samples. The predicted replication conditional means and variances show an excellent fit to the nonparametric estimates. The estimated non-null proportion is ${\hat{\pi }}_{2}=0.012$, indicating that about 1.2% of the pruned SNPs are in the large effect class. Thus, in terms of the proportion of large effect SNPs in pruned data, SZ is almost fifteen times more polygenic than CD. At the current effective sample size there are 15 SNPs with local fdr ≤ 0.05, or 1% of the estimated $N{\hat{\pi }}_{2}=$ 1,516 large-effect SNPs. The null standard deviation is estimated to be ${\hat{\sigma }}_{0}=1.01$, very close to the theoretical null. The standard deviation for large effects is ${\hat{\sigma }}_{2}=0.020$. Despite being more polygenic, large effect SNPs in SZ on average account for only 7% of the phenotypic variance accounted for by large effect SNPs in the CD data.

The standard deviation for small effects is ${\hat{\sigma }}_{1}=0.007$, and hence the empirical null variance is approximately ${\hat{\sigma }}_{0}^{2}+2\bar{p}\left(1-\bar{p}\right)n{\hat{\sigma }}_{1}^{2}=1.32$. Since ${\hat{\sigma }}_{1}>0$, as with CD there is a positive slope through the origin of the replication *z*-scores as a function of discovery *z*-scores (upper right panel of Fig 1) which scales with the size of the training sample (see S1 Fig). This is in contrast to what would be expected if the observed *z* scores were a mixture of true null (exactly zero) and non-null (non-zero) effects (S2 Fig), in which case there would be no positive slope through the origin.

### Finite Sample Prediction and False Discovery Rate

**Fig 2:**
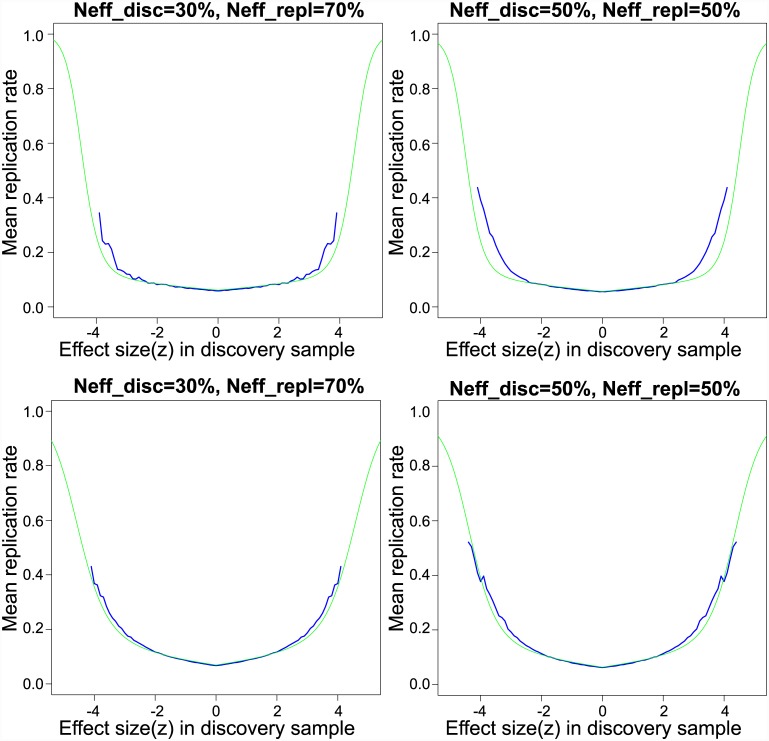
Empirical and model-based replication rates for schizophrenia. Empirical (black lines) and model-based (red lines) finite sample replication estimates. Left panel displays the average replication proportion conditional on discovery sample *z*-scores, for 30% of the overall sample apportioned to discovery sample, with the remainder apportioned to the replication sample. Red lines are computed from best fitting scale mixture of two normals. The middle panel displays the same for 50%, and the right panel for 70% of the overall sample apportioned to the training sample.

For the SZ data, parameter estimates from the scale mixture of normals model were used to compute the probability that a SNP will replicate given its observed training *z*-score, as given in Eq (15). Fig 2 displays the resampling-based replication rate and model-based replication probabilities for the CD and SZ meta-analyses, for resampling performed using 30% and 50% of the data in the training sample and the remainder in the replication sample. Fig 2 shows good agreement of the resampling-based replication rates with the mixture model-based replication probabilities for SZ. For CD, model-based replication probabilities underestimate the resampling-based replication rates in the tails, again due to excess kurtosis not captured by the two scale mixture components.

**Fig 3:**
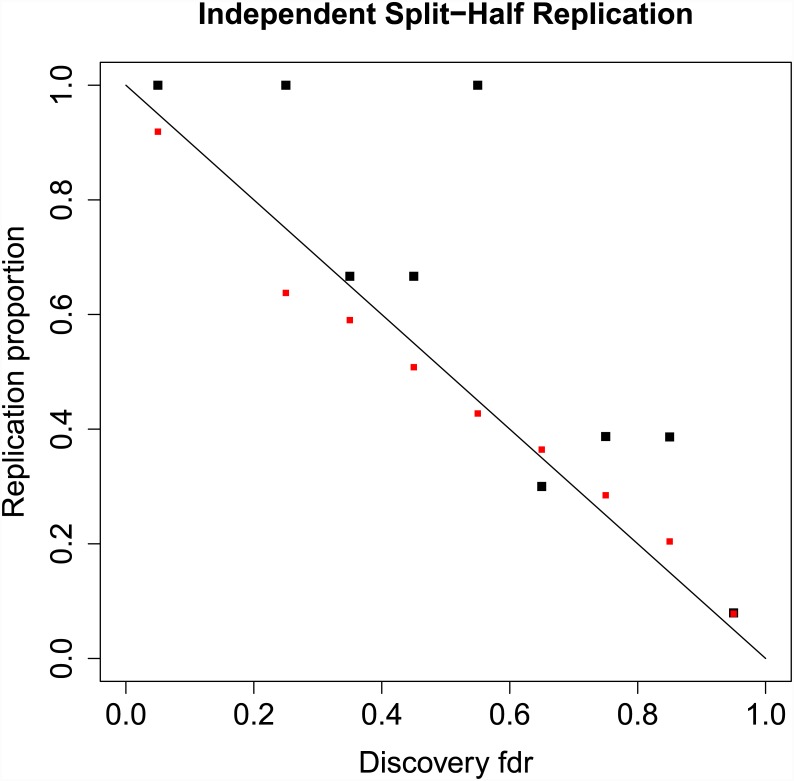
Replication proportions and predicted replication probabilities. Local fdr estimate are shown on the x-axis (binned from 0 to 1 in increments of 0.10), with discovery fdr computed on 26 randomly selected sub-studies in the PGC schizophrenia data consisting of 17,691 cases and 24,683 controls on *N* = 129,973 SNPs pruned to pairwise LD ≤ 0:20. For the independent replication sample we computed the meta-analysis *z*-scores using the remaining 26 studies, with 17,785 cases and 22,156 controls. Replication was defined as: (i) discovery and replication *z*-scores have same sign, and (ii) replication *z*-score associated with one-tailed *p*-value ≤ 0:05. Black squares show actual replication proportions for each bin, whereas red squares show mean predicted replication probabilities given in Eq (15).

The results displayed in Fig 2 do not constitute a true replication analysis, since the entire set of 52 studies was used to estimate the mixture model parameters. To assess true replication, we divided the sub-studies into disjoint “discovery” and “replication” samples. For the discovery sample, we computed the meta-analysis *z*-scores and local fdrs using summary statistics from 26 randomly selected sub-studies, consisting of 17,691 cases and 24,683 controls on the same set of *N* = 129,973 SNPs pruned to pairwise LD ≤ 0.20. For the replication sample we computed the meta-analysis *z*-scores using the remaining 26 studies, with 17,785 cases and 22,156 controls. We defined replication for a locus as having a one-sided replication *p*-value ≤ 0.05 and discovery and replication *z*-scores having the same sign. Other definitions of replication can be easily implemented. Replication proportions and mean predicted replication probabilities using Eq (15) are displayed in Fig 3. While replication proportions are noisy due to small numbers of SNPs in most fdr bins ([0, 0.1): 6, [0.1, 0.2): 0, [0.2, 0.3): 1, [0.3, 0.4): 3, [0.4, 0.5): 3, [0.5, 0.6): 7, [0.6, 0.7): 10, [0.7, 0.8): 31, [0.8, 0.9): 132, [0.9, 1.0]: 129,780), they generally track the predicted replication probabilities, showing some evidence, however, that predicted replication probabilities may be somewhat lower than actual replication rates. A downward bias in predicted replication probabilities could be caused by under-fitting the extreme tails of the distribution; this could potentially be rectified by adding one or more normal mixtures over the current two.

### Proportion of Posterior Heritability and Power

For a given threshold it is possible to estimate the proportion of posterior expected additive variance explained by SNPs selected using a given significance threshold. Let *c* > 0 be a given significance threshold, so that any SNP |*Z*| ≥ *c* is declared significant. Let *z*_*i*_ and *δ*_*i*_ denote the Wald statistic of the *i*th SNP with effect size *δ*_*i*_ as given in Eq (3). The proportion of genetic variance explained by these SNPs based on the scale mixture of two normals model is approximately

$$\begin{array}{c} h_{c}^{2}\approx \frac{\sum_{|z_{i}|\geq c}\hat{\text{E}}\left\{\delta_{i}^{2}|Z_{i}=z_{i}\right\}}{\sum_{i=1}^{N}\hat{\text{E}}\left\{\delta_{i}^{2}|Z_{i}=z_{i}\right\}} \end{array}$$ (1)

where $\hat{\text{E}}\left\{\delta_{i}^{2}|Z_{i}=z_{i}\right\}$ is estimated via Eq (13), substituting estimates $\hat{\theta }$ for ***θ***. This estimate relies on the assumption that the average LD of SNPs declared significant is roughly the same as the average LD of all SNPs, or that SNPs are first pruned for approximate independence. We can also modify Eq (1) to give the proportion of variance due to large effects accounted for by SNPs declared significant

$$\begin{array}{c} h_{c,1}^{2}\approx \frac{\sum_{|z_{i}|\geq c}{\hat{\text{E}}}_{1}\left\{\delta_{i}^{2}|Z_{i}=z_{i}\right\}}{\sum_{i=1}^{N}{\hat{\text{E}}}_{1}\left\{\delta_{i}^{2}|Z_{i}=z_{i}\right\}} \end{array}$$ (2)

where E_1_ denotes the posterior expectation due to large effects [27].

Using the parameters from the model-based fits, we can compute power for discovery when SNPs are declared non-null based on local fdr or *p*-value cut-offs. It is convenient to express power as the proportion of the genetic variance due to additive effects discovered for a given threshold. For example, the 45 SNPs with fdr ≤ 0.05 in the pruned CD data account for 55% of the genetic variance due to additive common effects in the pruned sample, including both large and small replicating effects. However, these loci account for 83% of variance due to large effects alone. Power estimates for CD are conservative, since the tails of the distribution are somewhat underestimated by the mixture of two normals model. In the SCZ data, the 15 SNPs with fdr ≤ 0.05 account for 3% of the variance due to the additive effects of all common variants, but 34% of the variance due to large effects alone. The difference in power between the two disorders is due to the more polygenic nature of SZ compared to CD, combined with its much smaller average size per “large-effect” SNP.

**Fig 4:**
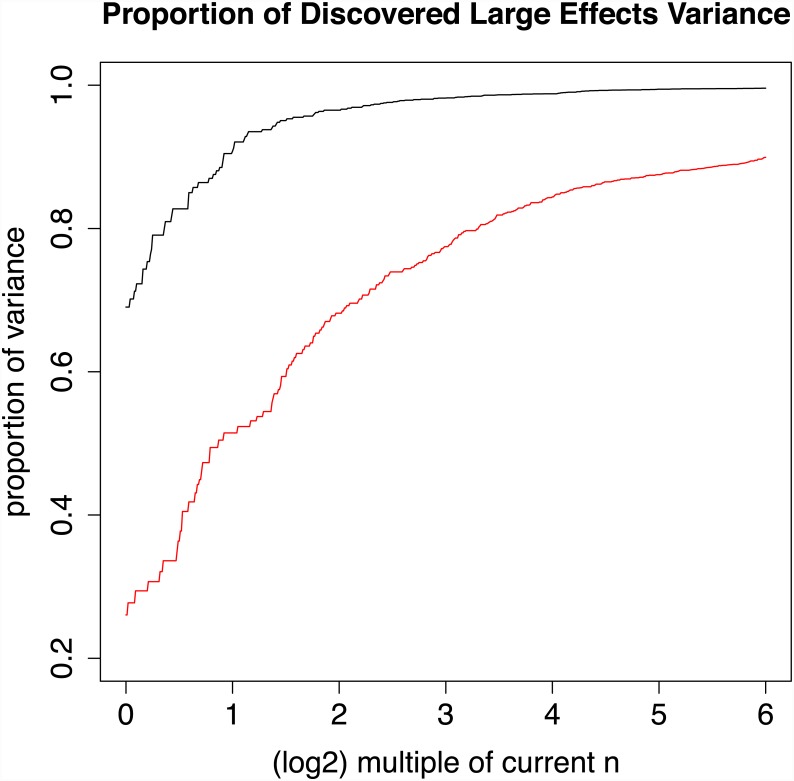
Power as a multiple of current effective sample size for Crohn’s disease and schizophrenia. Black line displays estimated proportion of additive genetic variance due to large effects for CD data, using a GWAS significance threshold of 5 × 10^−8^, current sample size (log_2_ 32 = 0) to 64 times current sample size (log_2_ 32 = 5). Red line displays same quantities for schizophrenia data.

Fig 4 displays the power for discovery for a genome-wide significance threshold of *p* ≤ 5 × 10^−8^ for increasing effective sample sizes for both CD and SZ. The *z*-scores are corrected using λ_GC_ as defined in [28]. For example, for CD the current sample size results in 69% of the variance due to large effect discovered; doubling the sample size for CD would result in the discovery of almost 91%. In contrast, using the same threshold for SZ, the current sample size uncovers SNPs accounting for only 26% of the large effect variance. The sample size would have to be increased 32-fold to detect 90% of the variance due to large effects, despite the fact that the current sample size of the SZ study is already much larger than that of the CD. One reason for the slow increase in power is that the median of the *z*^2^ distribution is inflated by both small and large effect variances, and hence the genomic inflation factor λ_GC_ [28] grows as a function of effective sample size *n*.

### Simulations

We conducted a series of Monte Carlo simulation studies to evaluate the performance of the fitting algorithm under different values of the parameters and departures from the standard meta-analysis assumptions (I)-(III) (see Models section) on the nonparametric estimates given in Eq (16) as well as the scale mixture of normals model parameters ***θ*** = {*π*_1_, *σ*_0_, *σ*_1_, *σ*_2_}, where *π*_1_ is the proportion of small effects, and *σ*_0_, *σ*_1_, and *σ*_2_ are the standard deviations of the null, small effect, and large effect (normal) distributions, respectively, as given in Eq (11). The results of these simulations are presented in S3–S7 Figs in the S1 Text section.

The estimates $\hat{\theta }$ produced by minimizing the quadratic estimating equations given in Eq (18) are in general unbiased and exhibit low variability across iterations of the simulations for a wide variety of parameter settings (S3 Fig). In S4 Fig and S6 Fig, we show the impact of large random departures from assumption (II): common minor allele frequencies (MAFs) across sub-studies; large departures from assumptions (I) and (III) will have similar effects. In these simulations, the estimated non-null proportion ${\hat{\pi }}_{2}$ is largely unaffected, ${\hat{\sigma }}_{0}$ is slightly elevated, and ${\hat{\sigma }}_{1}$ and ${\hat{\sigma }}_{2}$ are substantially decreased from the true values. In the scenario of large random departures from the overall mean values of the parameters, a random effects meta-analysis is more appropriate [29].

In the S1 Text section we also present simulations demonstrating the effects of LD on the distribution of effect sizes produced from massively univariate regression analyses typical of most GWAS. As described in [13] and in the S1 Text, LD “blurs” effect sizes from multiple loci, i.e., the expected effect size of a given locus produced from a univariate regression is a weighted sum of effects from all loci it is in non-zero LD with.

## Discussion

In this paper we derive the connection between a simple (four parameter) scale mixture of two normals model for effect size distributions and several quantities of interest in genome-wide studies. Specifically, parameter estimates from such a mixture model can be used to compute the proportion of genotyped SNPs with “large” effects, the local false discovery rate, probability of replication in a *de novo* sample, and power for discovery expressed as proportion of chip heritability explained for a given sample size and significance threshold. Effect size estimates can also be used for applications such as computation of polygenic risk for disorders (see S1 Text for how posterior effect sizes can be used in this fashion). Estimated effect sizes are shrunk empirically via the resampling process, and hence are free from the Winner’s Curse.

Direct observation demonstrates that for the schizophrenia GWAS data the scale mixture of two normals model provides a very good fit to nonparametric replication *z*-scores. The fit to the Crohn’s disease data is not as good, since the tails of the distribution are underestimated. This can be remedied by adding more components to the scale mixture, with two additional parameters per component. Derivations of local fdr, replication probabilities, and power presented in the Models section can be extended to more than two components. Underestimating the tails of the effect size distribution leads to conservative estimates of replication probabilities, local fdr, and power for discovery.

An interesting aspect of using the resampling-based fitting procedure is the ability to separate the null standard deviation *σ*_0_ from the standard deviation *σ*_1_ of small but replicating effects, which are confounded in non-resampling based fitting algorithms for mixture models employing the “empirical null” (e.g., [23]). Small replicating effects which scale with sample size could potentially be due to residual population stratification or to weak yet pervasive LD with causal effects. The later case would suggest that weak LD with causal variants may be a significant source of variation in tests statistics, as discussed in [13]. (Note, however, that [13] does not model the distribution of effect sizes and hence does not assess differential effects of LD on null vs. non-null loci.) An important consequence of the presence of small and large effects whose variances scale linearly with effective sample size is that the genomic inflation factor λ_GC_ [28] also grows as a function of sample size. It has been argued that the distribution of non-null effects substantially accounts for the observed genomic inflation in large GWAS [15, 26]. While our results are consonant with this fact, we here make a more fine-grained distinction between genomic inflation due to small and that due to large replicating effects. To the degree that small effect inflation is considered spurious, performing no genomic inflation control whatsoever would appear to be overly liberal.

A weakness of the resampling procedure is that the quadratic estimating equations do not produce accurate confidence intervals for parameter estimates. This is due to the complicated correlation structure among terms in the estimating equations induced by the presence of LD in the SNPs and by the overlap in randomly resampled estimates. In theory it is possible to obtain the overall effective degrees of freedom of the estimates by computing the mean induced correlation which can then be used to adjust the length of standard confidence intervals. Non-resampling based mixture model algorithms also exist that estimate the non-null distribution using likelihood-based flexible regression fits (e.g., see [23]), and we are currently developing a fully Bayesian alternative that models the non-null distribution as a location mixture of B-spline densities with mixture weights that can depend on LD and multiple genic annotation categories. These non-resampling based algorithms can provide accurate confidence intervals for parameters assuming the data are first pruned for approximate independence.

Another disadvantage of the proposed algorithm is that splitting studies into disjoint training and replication sets leads to lower power to estimate the non-null component of the mixture when the sample size is small, where “small” depends on the level of polygenicity and the average size for non-null effect. As such, the resampling-based algorithm depends on a fairly sizable signal in the GWAS data so that the parameters *π*_2_ and *σ*_2_ can be estimated.

In general, it is crucial to consider the impact of LD on the massively univariate regression estimates common to standard GWAS analyses, since regression weights $\hat{b}$ have expectations that depend heavily on the LD structure (see S1 Text). In particular, the expectation of ${\hat{b}}_{i}$ is equal to the causal effect of the *i*th SNP plus a weighted sum of all the causal effects it is in LD with (see S1 Text for details and [13]). The effects of LD on nonparametric estimates of the effect size distribution, and hence also on estimates of parameters from the scale-mixture of normal model, can be profound. Simulations (S5 Fig and S7 Fig) also show an over 20-fold increase in *π*_2_ estimates from the generative model compared to the distribution of observed *z*-scores. These simulations present a worst-case scenario for inflation of *π*_2_: no pruning, all causal effects are in the middle of large LD blocks, and every other SNP in the block is null. In reality, LD blocks containing functional genomic regions appear to have a higher proportion of non-null effects than can be explained by inflation of statistics due to LD alone [17]. LD pruning would also lower the estimate of *π*_2_ much closer to the causal proportion. The efficient and accurate handling of the effects of LD on effect size distributions is an area of active research.

## Models

### Association Statistics

For the *j*th subject, *j* = 1,…, *n*, the genotype-phenotype data consist of {***x***_*j*_, *y*_*j*_}, where ***x***_*j*_ is the vector of mean-centered allele counts from *N* assayed bi-allelic loci (SNPs) and *y*_*j*_ either is a continuous response, or *y*_*j*_ ∈ {0, 1} for case-control data, where 0 denotes control and 1 denotes case status. Let **X** = (**ξ**_1_, …, **ξ**_*N*_) be the *n* × *N* matrix of allele counts, where **ξ**_*i*_ is the *n* × 1 column vector of allele counts for the *i*th genetic locus. (Thus, the *j*th row of **X** is given by $x_{j}^{T}$, where superscript *T* denotes the transpose of a vector or matrix.) Under Hardy-Weinberg Equilibrium (HWE), the elements of **ξ**_*i*_ are distributed as centered binomial random variables, *Bin*(2, *p*_*i*_) − 2*p*_*i*_, where *p*_*i*_ is the effect allele frequency for the *i*th SNP. In the sequel, we assume *p*_*i*_ is known, ignoring uncertainty due to estimation, which has no impact on the asymptotic results.

Let ${\hat{b}}_{i}$ denote the regression coefficient of **ξ**_*i*_ on the outcome vector **y** = (*y*_1_, …, *y*_n_)*^T^*. In this paper, we assume that the vector of regression coefficients $\hat{b}={\left({\hat{b}}_{1},\ldots ,{\hat{b}}_{N}\right)}^{T}$ is produced using massively univariate linear (for continuous) or logistic (for dichotomous) regressions. However, the resampling methodology described below is applicable to any regression coefficient estimates $\hat{b}$, including, for example, *best linear unbiased predictors* (BLUPs) from random-effects models [3, 30, 31], which may provide better localization of effects. We describe the effects of LD on univariate estimates $\hat{b}$ both analytically and in Monte Carlo simulations in the S1 Text.

The regression coefficient estimates $\hat{b}$ are used to produce an *N*-dimensional vector of Wald test statistics (“*z*-scores”)

$$\begin{array}{c} z≃\sqrt{n}C\hat{b}, \end{array}$$

where **C** is an *N* × *N* diagonal matrix and ≃ denotes asymptotic equality as the effective sample size *n* goes to infinity. The diagonal entries $c_{ii}=\sqrt{2p_{i}\left(1-p_{i}\right)/\sigma_{i}^{2}}$, where *p*_*i*_ is the effect allele frequency for the *i*th SNP and *σ*_*i*_ is the residual standard deviation (for linear regression) or equal to 1 (for logistic regression). Thus

$$\begin{array}{ccc} z & ≃ & \sqrt{n}C\hat{b}\\ & = & \sqrt{n}\;\text{diag}\left\{\sqrt{\frac{2p_{i}\left(1-p_{i}\right)}{\sigma_{i}^{2}}}\right\}\hat{b}\\ & = & \sqrt{n}\delta +\omega \end{array}$$ (3)

where ***δ*** = (*δ*_1_, …, *δ*_*N*_)^*T*^ and ***ω*** = (*ω*_1_, …, *ω*_*N*_)^*T*^ are *N*-dimensional vectors such that

$$\begin{array}{cc} \delta_{i} & =\sqrt{\frac{2p_{i}\left(1-p_{i}\right)}{\sigma_{i}^{2}}}\;\text{E}\left\{{\hat{b}}_{i}\right\}\\ & =\sqrt{2p_{i}\left(1-p_{i}\right)}\;\frac{b_{i}}{\sigma_{i}} \end{array}$$ (4)

and $\omega_{i}∼\text{N}\left(0,\sigma_{0}^{2}\right)$. Here, *b*_*i*_ denotes the expectation $\text{E}\{{\hat{b}}_{i}\}$, and normality of ***ω*** follows from a large sample approximation (see S1 Text). We assume that the effect sizes are exchangeable with *δ*_*i*_ ∼ *g*(*δ*_*i*_), where *g* is an (unknown) marginal density. The theoretical value of the variance $\sigma_{0}^{2}=1$; however, $\sigma_{0}^{2}$ may be greater than 1 in the presence of the population substructure such as cryptic relatedness [28], and in the model fitting algorithm described below $\sigma_{0}^{2}$ is estimated from the data. In the remainder of the paper, we define the *effect size* of the *i*th SNP as $\delta_{i}=\sqrt{2p_{i}\left(1-p_{i}\right)}b_{i}/\sigma_{i}$.

Often the data available from large GWAS meta-analyses are the *z*-scores from the individual sub-studies, rather than the full genotypic and phenotypic data. In this scenario, it is possible to use the proposed re-sampling based algorithm using *z*-scores from the individual studies. Suppose the data (***X***, ***y***) are partitioned into *K* disjoint independent samples (sub-studies) {(***X***_*k*_,***y***_*k*_)∣*k* = 1, …, *K*}, each with effective sample size *n*_*k*_. The *k*th sub-study is used to compute an *N*-dimensional vector of SNP regression weights ${\hat{b}}_{k}$. The *z*-scores from each sub-study are given by

$$\begin{array}{c} z_{k}≃\sqrt{n_{k}}C_{k}{\hat{b}}_{k}, \end{array}$$

where $C_{k}=\text{diag}\{\sqrt{2p_{k,i}\left(1-p_{k,i}\right)/\sigma_{k,i}^{2}}\;\}$ is an *N* × *N* diagonal matrix and $\sigma_{k,i}^{2}$ is the residual variance in the *i*th regression (for continuous outcomes) or 1 (for logistic regression on discrete outcomes). If for *k* = 1, …, *K*, *i* = 1, …, *N*, we assume (I) $\sigma_{k,i}^{2}=\sigma_{i}^{2}$; (II) effect allele frequencies *p*_*k*,*i*_ = *p*_*i*_; and (III) $b_{k,i}=\text{E}\left\{{\hat{b}}_{k,i}\right\}=b_{i}$; then, the diagonal entries $c_{k,ii}=c_{ii}=\sqrt{2p_{i}\left(1-p_{i}\right)/\sigma_{i}^{2}}$ and

$$\begin{array}{lll} z_{k} & ≃ & \sqrt{n_{k}}C_{k}{\hat{b}}_{k}\\ & = & \sqrt{n_{k}}\delta +\omega_{k}\\ & = & \delta_{k}+\omega_{k}, \end{array}$$

where $\delta_{k}\equiv \sqrt{n_{k}}\delta$ and ***δ*** = (*δ*_1_, …,*δ*_*N*_)^*T*^, with $\delta_{i}=\sqrt{2p_{i}\left(1-p_{i}\right)}\left(\beta_{i}/\sigma_{i}\right)$. Thus, ***δ***_*k*_ differs across sub-studies only in the multiplicative factor $\sqrt{n_{k}}$. Assumptions (I)–(III) should be approximately valid if the sub-studies can be considered random draws from the same population. Note, assumptions (I)–(III) are also necessary for meta-analyses to be valid; hence, the assumptions necessary for the random partitioning algorithm proposed below are precisely the standard assumptions used in GWAS meta-analyses [32]. Alternatively, if there are random departures from assumptions (I)–(III), a meta-analysis treating sub-study *z*-scores as random effects could be performed [29].

If the sub-study *z*-scores {**z**_1_, …,**z**_*K*_} are given, the overall meta-analysis *z*-scores can be computed as a weighted sum [32]

$$\begin{array}{c} z=\frac{\sum_{k=1}^{K}\sqrt{n_{k}}z_{k}}{\sqrt{\sum_{k=1}^{K}n_{k}}}=\sqrt{n}\delta +\omega , \end{array}$$ (5)

where $n=\sum_{k=1}^{K}n_{k}$ and $\omega =\sum_{k=1}^{K}\sqrt{n_{k}}\omega_{k}/\sqrt{n}$ and again $w_{i}∼\text{N}\left(0,\sigma_{0}^{2}\right)$. In both the Crohn’s disease and the schizophrenia GWAS examples, meta-analysis *z*-scores are produced using fixed-effects methods, as in their original papers [1, 26].

### Posterior Expectations and Variances

The *N* × 1 vector of effect sizes ***δ*** is of fundamental interest in GWAS analyses, closely related to power for discovery, proportion of chip heritability discovered, the probability that a SNP is null given its observed *z*-score, and polygenic risk estimation. As above, let ***z*** = (*z*_1_, …,*z*_*N*_)^*T*^ denote the *N*-dimensional vector of *z*-scores, where *n* is the effective sample size of the study. From Eq (3), these *z*-scores are derived from the simple measurement model $z=\sqrt{n}\delta +\omega$, where ***δ*** is the *N* × 1 are random draws from an unknown effect size distribution independent of the $\omega_{i}\overset{iid}{∼}\text{N}\left(0,\sigma_{0}^{2}\right)$.

Since the *δ*_*i*_ are not observed directly, we are interested in the marginal posterior distributions of *δ*_*i*_ given the observed test statistic *z*_*i*_. For many uses it is sufficient to obtain the posterior means ($\text{E}\{\sqrt{n}\delta_{i}|z_{i}\}$) and variances ($\text{Var}\{\sqrt{n}\delta_{i}|z_{i}\}$), for *i* = 1, …, *N*. By Theorem 11.1 of [23] (p. 221), these are given by

$$\begin{array}{cc} \text{E}\{\sqrt{n}\delta_{i}|z_{i}\} & =z_{i}+\sigma_{0}^{2}\frac{d}{dz}\log\left\{f\left(z_{i}\right)\right\},\\ \text{Var}\{\sqrt{n}\delta_{i}|z_{i}\} & =\sigma_{0}^{2}\left[1+\sigma_{0}^{2}\frac{d^{2}}{dz^{2}}\log\left\{f\left(z_{i}\right)\right\}\right], \end{array}$$ (6)

where *f*(*z*_*i*_) is the common marginal probability density function (pdf) of the *z*_*i*_ and $\sigma_{0}^{2}$ is the variance of *ω*_*i*_. This result is quite general, essentially requiring only that *δ*_*i*_ and *ω*_*i*_ are independent and $\omega_{i}∼\text{N}\left(0,\sigma_{0}^{2}\right)$ [23].

### Two-Groups Mixture Model

A commonly employed Bayesian framework assumes that some proportion of the tests are generated under the null hypothesis (i.e., *δ*_*i*_ ≈ 0) and that the complement are generated under the non-null hypothesis (i.e., *δ*_*i*_¬≈0) [27]. To formalize this model, let (*Z*_*i*_, *H*_*i*_) be exchangeable random variables, *i* = 1, …,*N*, where as usual *Z*_*i*_ denotes the test statistic for the *i*th test, and *H*_*i*_ ∼ *Bernoulli*(*π*_2_) is an indicator of whether the *i*th test is null (*H*_*i*_ = 1) or non-null (*H*_*i*_ = 2), and hence *π*_2_ denotes the proportion of non-null effects, i.e., the *a priori* probability that a given hypothesis test is non-null. The marginal density of *Z*_*i*_ is given by

$$\begin{array}{c} f\left(z_{i}\right)=\pi_{1}f_{1}\left(z_{i}\right)+\pi_{2}f_{2}\left(z_{i}\right), \end{array}$$ (7)

where *π*_1_ = 1 − *π*_2_ is the null proportion, *f*_1_ is the null density, and *f*_2_ is the non-null density. Under the assumptions following Eq (3), the non-null density *f*_2_ is the convolution of a normal density with mean zero and variance $\sigma_{0}^{2}$, denoted by $\phi (\cdot |0,\sigma_{0}^{2})$, with the (as yet) unspecified non-null density *g* of *δ*.

The two-group mixture model given by Eq (7) is the foundation for the Bayesian interpretation of the false discovery rate [19, 33]. In particular, Efron [19] defined the *local false discovery rate* (fdr) as the posterior probability that *H*_*i*_ = 0 given *Z*_*i*_ = *z*_*i*_. By an application of Bayes’ Rule to Eq (7), the fdr is derived as

$$\begin{array}{ccc} \text{fdr}(z_{i}) & = & \text{Pr}(H_{i}=0|Z_{i}=z_{i})\\ & = & \frac{\pi_{1}f_{1}\left(z_{i}\right)}{f(z_{i})}. \end{array}$$ (8)

The *local true discovery rate* for the *i*th SNP is then defined simply as *tdr*(*z*_*i*_) = 1 − *fdr*(*z*_*i*_), the posterior probability that an effect is non-null given its observed test statistic *z*_*i*_. Local fdr can be used as a thresholding technique by selecting SNPs corresponding to fdr(*z*_*i*_)≤ *α* for some choice of cut-off, say *α* ≤ 0.05, or equivalently, selecting those SNPs for which tdr(*z*)>1 − *α*.

There is a close connection between Eq (6) and the fdr defined in Eq (8). By Corrollary 11.3 of [23] (p. 223), these are given by

$$\begin{array}{cc} \text{E}\{\sqrt{n}\delta_{i}|z_{i}\} & =-\frac{d}{dz}\log\left\{\text{fdr}\left(z_{i}\right)\right\}\\ \text{Var}\{\sqrt{n}\delta_{i}|z_{i}\} & =-\frac{d^{2}}{dz^{2}}\log\left\{\text{fdr}\left(z_{i}\right)\right\}. \end{array}$$ (9)

#### Scale mixture of normals model

We present a simple scale mixture of two normals model for the marginal density *g* of *δ*_*i*_

$$\begin{array}{c} g\left(\delta_{i}\right)=\pi_{1}\phi \left(\delta_{i}|0,\sigma_{1}^{2}\right)+\pi_{2}\phi \left(\delta_{i}|0,\sigma_{1}^{2}+\sigma_{2}^{2}\right), \end{array}$$ (10)

This model posits that effects come from a normal distribution $N(0,\sigma_{1}^{2})$ with probability *π*_1_ or from a normal distribution with larger variance, $N(0,\sigma_{1}^{2}+\sigma_{1}^{2})$ with probability *π*_2_ = 1 − *π*_1_. If $\sigma_{1}^{2}=0$, then $\phi (z_{i}|0,\sigma_{1}^{2})$ is a point mass (indicator function) at zero, i.e., effects drawn from this distribution are always exactly zero, corresponding to the null hypothesis *H*_*i*_: *δ*_*i*_ = 0. More generally, if $\sigma_{1}^{2}\geq 0$ this corresponds to a mixture of “small” and “large” effects, which includes zero and small non-zero effects as a special case. Large values of |*δ*_*i*_| will have a higher posterior probability of coming from the distribution with larger variance. From Eq (3), $z_{i}\approx \sqrt{n}\delta_{i}+\omega_{i}$, where $\omega_{i}∼\text{N}\left(0,\sigma_{0}^{2}\right)$ is independent of *δ*_*i*_. The marginal density of *Z*_*i*_ is thus given by

$$\begin{array}{ccc} f(z_{i}) & = & \pi_{1}\phi \left(z_{i}|0,\sigma_{0}^{2}+2np_{i}\left(1-p_{i}\right)\sigma_{1}^{2}\right)+\\ & & \pi_{2}\phi \left(z_{i}|0,\sigma_{0}^{2}+2np_{i}\left(1-p_{i}\right)\left[\sigma_{1}^{2}+\sigma_{2}^{2}\right]\right). \end{array}$$ (11)

Note, this scale mixture of normals is closely related to the model given in [10]. For a good discussion of mixture models and Bayesian selection in the context of genetic effect size distributions, see [7].

An advantage of the scale mixture of two normals model is its computational tractability. Model fitting involves estimation of only four parameters. Moreover, it is relatively straightforward to use estimates of these parameters to compute other quantities of interest. For example, we can express the fdr as

$$\begin{array}{cc} \text{fdr}(z_{i}) & =\text{Pr}(\delta_{i}\approx 0|z_{i})\\ & =\frac{\pi_{1}\phi \left(z_{i}|0,\sigma_{0}^{2}+2np_{i}\left(1-p_{i}\right)\sigma_{1}^{2}\right)}{\pi_{1}\phi \left(z_{i}|0,\sigma_{0}^{2}+2np_{i}\left(1-p_{i}\right)\sigma_{1}^{2}\right))+\pi_{2}\phi \left(z_{i}|0,\sigma_{0}^{2}+2np_{i}\left(1-p_{i}\right)\left[\sigma_{1}^{2}+\sigma_{2}^{2}\right]\right)}, \end{array}$$ (12)

Here, fdr refers to the posterior probability of being a “small” effect, which includes zero effects as a sub-case ($\sigma_{1}^{2}=0$). We can also derive the posterior expectations and variances of the effect sizes given the *z*-scores in terms of the mixture model. Let $\theta =\{\pi_{1},\sigma_{0}^{2},\sigma_{1}^{2},\sigma_{2}^{2}\}$, and let *μ*(*z*_*i*_, *n*, *p*_*i*_∣***θ***) denote the posterior expectation of $\sqrt{n}\delta_{i}$ given *z*_*i*_. Using the properties of conditional normal distributions and the fact that *fdr*(*z*_*i*_) = *P*(*H*_*i*_ = 1|*Z*_*i*_ = *z*_*i*_) and *tdr*(*z*_*i*_) = *P*(*H*_*i*_ = 2|*Z*_*i*_ = *z*_*i*_),

$$\begin{array}{cc} \mu (z_{i},n,p_{i}|\theta ) & \equiv \text{E}\{\sqrt{n}\delta_{i}|Z_{i}=z_{i}\}\\ & =\left(\frac{2np_{i}\left(1-p_{i}\right)\sigma_{1}^{2}}{\sigma_{0}^{2}+2np_{i}\left(1-p_{i}\right)\sigma_{1}^{2}}\right)\text{fdr}\left(z_{i}\right)+\left(\frac{2np_{i}\left(1-p_{i}\right)\left[\sigma_{1}^{2}+\sigma_{2}^{2}\right]}{\sigma_{0}^{2}+2np_{i}\left(1-p_{i}\right)\left[\sigma_{1}^{2}+\sigma_{2}^{2}\right]}\right)\text{tdr}\left(z_{i}\right). \end{array}$$ (13)

Moreover, the posterior variance *σ*^2^(*z*_*i*_, *n*, *p*_*i*_∣***θ***) of $\sqrt{n}\delta_{i}$ given *z*_*i*_ is given by

$$\begin{array}{cc} \sigma^{2}\left(z_{i},n,p_{i}|\theta \right) & \equiv \text{Var}\{\delta_{i}|Z_{i}=z_{i}\}\\ & =\sigma_{0}^{2}+\sigma_{0}^{4}[\frac{z_{i}^{2}-\left(\sigma_{0}^{2}+2np_{i}\left(1-p_{i}\right)\sigma_{1}^{2}\right)}{{\left(\sigma_{0}^{2}+2np_{i}\left(1-p_{i}\right)\sigma_{1}^{2}\right)}^{2}}\text{fdr}\left(z_{i}\right)\\ & \;+\frac{z_{i}^{2}-\left(\sigma_{0}^{2}+2np_{i}\left(1-p_{i}\right)\left[\sigma_{1}^{2}+\sigma_{2}^{2}\right]\right)}{{\left(\sigma_{0}^{2}+2np_{i}\left(1-p_{i}\right)\left[\sigma_{1}^{2}+\sigma_{2}^{2}\right]\right)}^{2}}\text{tdr}\left(z_{i}\right)\\ & \;\;-z_{i}^{2}{\left(\frac{\text{fdr}(z_{i})}{\sigma_{0}^{2}+2np_{i}\left(1-p_{i}\right)\sigma_{1}^{2}}+\frac{\text{tdr}(z_{i})}{\sigma_{0}^{2}+2np_{i}\left(1-p_{i}\right)\left[\sigma_{1}^{2}+\sigma_{2}^{2}\right]}\right)}^{\;2}]. \end{array}$$ (14)

We can also use the mixture model parameters to compute the finite-sample probability that the *i*th SNP will replicate given its observed *z*-score *z*_*i*_. Suppose we have a training study with effective sample size *n* producing *z*-score *Z*, and a replication study with effective sample size *n*_*r*_ and *z*-score *Z*_*r*_. We define the replication for the *i*th SNP as (i) sign(*Z*_*i*_) = sign(*Z*_*r*,*i*_); and (ii) |*Z*_*i*_| ≥ *c*_*α*_ for some significance threshold *c*_*α*_. For example, *c*_*α*_ = 1.64 corresponds to a one-sided *p* = 0.05. Using the properties of conditional normal distributions we can write this probability as

$$\begin{array}{c} \text{P}(|Z_{\text{r},i}|\geq c_{\alpha }\;\text{and}\;\text{sign}\left(Z_{\text{r},i}\right)=\text{sign}\left(Z_{i}\right)|Z_{i}=z_{i})\;\\ =\Phi \left(-c_{\alpha }|\mu_{\text{r},0},\sigma_{\text{r},0}^{2}\right)\text{fdr}\left(z_{i}\right)+\Phi \left(-c_{\alpha }|\mu_{\text{r},1},\sigma_{\text{r},1}^{2}\right)\text{tdr}\left(z_{i}\right), \end{array}$$ (15)

where Φ(⋅ ∣*μ*,*σ*^2^) is the cumulative distribution function (cdf) of the normal distribution with mean *μ* and variance *σ*^2^, and

$$\begin{array}{cc} \mu_{\text{r},0} & =-\left(\frac{\sqrt{nn_{\text{r}}}2p_{i}\left(1-p_{i}\right)\sigma_{1}^{2}}{\sigma_{0}^{2}+2np_{i}\left(1-p_{i}\right)\sigma_{1}^{2}}\right)\left|z_{i}\right|,\\ \sigma_{\text{r},0}^{2} & =\sigma_{0}^{2}+2n_{\text{r}}p_{i}\left(1-p_{i}\right)\sigma_{1}^{2}-\frac{nn_{\text{r}}{\left(2p_{i}\left(1-p_{i}\right)\sigma_{1}^{2}\right)}^{2}}{\sigma_{0}^{2}+2np_{i}\left(1-p_{i}\right)\sigma_{1}^{2}},\\ \mu_{\text{r},1} & =-\left(\frac{\sqrt{nn_{\text{r}}}2p_{i}\left(1-p_{i}\right)\left[\sigma_{1}^{2}+\sigma_{2}^{2}\right]}{\sigma_{0}^{2}+2np_{i}\left(1-p_{i}\right)\left[\sigma_{1}^{2}+\sigma_{2}^{2}\right]}\right)\left|z_{i}\right|,\\ \sigma_{\text{r},1}^{2} & =\sigma_{0}^{2}+2n_{\text{r}}p_{i}\left(1-p_{i}\right)\left[\sigma_{1}^{2}+\sigma_{2}^{2}\right]-\frac{nn_{\text{r}}{\left(2p_{i}\left(1-p_{i}\right)\left[\sigma_{1}^{2}+\sigma_{2}^{2}\right]\right)}^{2}}{\sigma_{0}^{2}+2np_{i}\left(1-p_{i}\right)\left[\sigma_{1}^{2}+\sigma_{2}^{2}\right]}. \end{array}$$

For large values of *n*_*r*_, if $\sigma_{1}^{2}\approx 0$ the finite sample replication rate given in Eq (15) reduces to *tdr*(*z*_*i*_). Thus, an accurate model-based finite-sample replication prediction provides empirical justification for using the estimated *fdr*(*z*_*i*_) as a cut-off providing accurate false discovery rate control.

#### Nonparametric estimates

Other authors have proposed estimating effect sizes via 10-fold cross-validation or bootstrapping [34, 35]. These approaches shrink effect sizes towards zero, to avoid the positive upward bias in estimation of effects due to selection or ranking. They demonstrate that, by selecting tests based on p-values from a random subsample of data and then estimating the effect sizes on the out-of-sample data, estimates of effect sizes are substantially less biased than the naive estimates that use the same data directly for selection and estimation of non-null effect sizes.

We take an approach related to the 10-fold cross-validation algorithm given in [34]. The algorithm we propose repeatedly and randomly partitions the sub-study *z*-scores into training and replication sets. In contrast to [34], at each iteration an approximately unbiased estimate of Eq (6) is constructed by binning all tests according to their training *z*-scores and averaging replication *z*-scores separately by bin. By randomly partitioning the data and averaging estimates across iterations, we obtain an estimate which is again approximately unbiased and which smooths out random deviations due to arbitrarily partitioning studies into “discovery” and “replication” samples.

More specifically, to obtain nonparametric estimates of *E*{*δ*_*i*_ ∣ *z*_*i*_}, the sub-studies are randomly partitioned into two groups *K* times. For each iteration *k* = 1, …, *K*, one group is labeled a *discovery* sample and the other is labeled a *replication* sample. Eq (5) is applied separately to each group to obtain independent meta-analysis training *z*-scores *Z*_*i*[*k*]_ and replication *z*-scores *Z*_*r*,*i*[*k*]_, for *i* = 1, …, *N*. The *Z*_*i*[*k*]_ are then binned into intervals $I_{m}=\left[-c+\left(m-1\right)h,-c+mh\right]$ of width *h* on the interval [−*c*, *c*) for fixed *c* > 0, where *m* = 1, …, *M*. Let $Z_{m[k]}=\left\{Z_{i[k]}:Z_{i[k]}\in I_{m}\right\}$, and let $n_{m[k]}=\text{card}\left\{Z_{m[k]}\right\}$, where “card” denotes the cardinality, or number of elements in the set. We compute the sample means ${\bar{Z}}_{\text{r},m[k]}=\left(1/n_{m[k]}\right)\sum_{i:Z_{i[k]}\in Z_{m[k]}}Z_{\text{r,}i[k]}$ and mean squares ${\bar{Z^{2}}}_{\text{r},m[k]}=\left(1/n_{m[k]}\right)\sum_{i:Z_{i[k]}^{2}\in Z_{m[k]}}Z_{\text{r,}i[k]}^{2}$ and average these across iterations *k*, to obtain smoothed estimates

$$\begin{array}{cc} {\bar{Z}}_{\text{r},m} & =\frac{1}{K}\sum_{k=1}^{K}{\bar{Z}}_{\text{r},m[k]}\\ {\bar{Z^{2}}}_{\text{r},m} & =\frac{1}{K}\sum_{k=1}^{K}{\bar{Z^{2}}}_{\text{r},m[k]},\;m=1,\ldots ,M. \end{array}$$ (16)

Under the assumption that subjects in the discovery and replication samples are independent, we have

$$\begin{array}{cc} \text{E}\{{\bar{Z}}_{\text{r},m}\} & =\text{E}\left\{Z_{\text{r}}|Z\in I_{m}\right\}=\sqrt{\frac{n_{\text{r}}}{n}}\text{E}\left\{\sqrt{n}\delta |Z\in I_{m}\right\},\\ \text{E}\{{\bar{Z^{2}}}_{\text{r},m}\} & =\text{E}\left\{Z_{\text{r}}^{2}|Z\in I_{m}\right\}=\frac{n_{\text{r}}}{n}\text{E}\left\{{\left[\sqrt{n}\delta \right]}^{2}|Z\in I_{m}\right\}+\sigma_{0}^{2}, \end{array}$$ (17)

where *n* and *Z* are the effective sample size and the *z*-score of the discovery sample, and *n*_*r*_ and *Z*_*r*_ are the effective sample size and the *z*-score of the replication sample. Eq (17) are linear transformations of *E*{*δ*∣*Z* = *z*} and *E*{*δ*^2^∣*Z* = *z*} and hence Eq (16) serve as nonparametric estimates of the first two moments of the effect sizes *δ* given training the *z*-scores.

### Estimation of Parameters

For a given model E(θ) and V(θ) for the distribution of effect size expectations and variances, we can estimate parameters ***θ*** by utilizing Eqs (6) and (17). Specifically, we enter the model-based predictions (dependent on parameters ***θ***) into quadratic estimating equations that solve for parameter estimates minimizing the differences between the empirical and model-based replication expectations and variances. For the scale mixture of normals model, Eqs (13) and (14) are entered into the quadratic equations.

$$\begin{array}{ccc} Q(\theta ) & = & \sum_{m=1}^{M}\sum_{\rho \in R}\left[{\left(\text{E}\left\{{\bar{Z}}_{\text{r},m}\right\}-\sqrt{\rho }\mu \left(Z_{m},n_{\rho },\bar{p}|\theta \right)\right)}^{2}+\right.\\ & & \;\;\;\;\;\left.{\left(\text{Var}\left\{Z_{\text{r},m}^{2}\right\}-\rho \sigma^{2}\left(Z_{m},n_{\rho },\bar{p}|\theta \right)-\sigma_{0}^{2})\right)}^{2}\right], \end{array}$$ (18)

where $\text{E}\{{\bar{Z}}_{\text{r},m}\}$ is the nonparametric posterior mean estimate of the *m*th bin $I_{m}$ given in Eq (17), $\text{Var}\left\{Z_{\text{r},m}^{2}\right\}=\text{E}\left\{{\bar{Z^{2}}}_{\text{r},m}\right\}-\text{E}{\left\{{\bar{Z}}_{\text{r},m}\right\}}^{2}$ is the nonparametric variance estimate, *Z*_*m*_ is the midpoint of $I_{m}$, $\bar{p}$ is the average effect allele frequency, and *ρ* = *n*_*r*,*ρ*_/*n*_*ρ*_ is the ratio of the effective sample size of the replication sample (*n*_*r*,*ρ*_) over the effective sample size of the discovery sample (*n*_*ρ*_). The advantage of varying *ρ* is the ability to observe the effects of changing sample size on the effect size distribution and finite-sample replication rates.

In the real applications below, we keep *ρ* = 0.5 for the Crohn’s disease data, and we vary *ρ* between 0.3 and 0.5 in the schizophrenia data. Monte Carlo simulations in the S1 Text section use split-half samples (*ρ* = 0.50). For all analyses, the bin width *h* was chosen such that there were *M* = 201 bins equally-spaced bins spanning the range of *z*-scores. The values for *c* are chosen to span the entire range of observed *z*-scores in the given analysis. For the Crohn’s disease example *c* = 17, for schizophrenia example *c* = 12, and in the simulations *c* = 10. Note, the mean allele frequency $\bar{p}$ is used in place of the actual frequency for computational efficiency. Actual values of *p*_*i*_ could be incorporated by binning with respect to effect allele frequency in addition to binning by discovery *z*-score; however, in practice this appears to have little effect on estimates.

Eq (18) is minimized over the parameter space ***θ*** using a simplex algorithm to produce estimated values $\hat{\theta }\equiv \left\{\hat{\pi_{1}},{\hat{\sigma_{0}}}^{2},{\hat{\sigma }}_{1}^{2},{\hat{\sigma }}_{2}^{2}\right\}$ that can then be used to estimate posterior effect sizes, the finite-sample probabilities that SNPs will replicate given their observed *z*-scores, and the local false discovery rate.

The resampling and fitting algorithm is available in R and Matlab scripts, along with code to generate synthetic sub-study GWAS *z*-scores, at https://sites.google.com/site/covmodfdr/.

## Supporting Information

S1 Text S1 Text Sections 1–2 provide statistical derivations of the effects of linkage disqequilibrium (LD) on massively univariate regression estimates from GWAS. Section 3 outlines how posterior effect size estimates from the mixture model could be used in polygenic risk score estimation. Section 4 provides details and results from Simulation Studies, as described in the main text. Section 5 gives the authorship list for the Psychiatric Genetics Consortium Schizophrenia Working Group.
(PDF)

S1 Fig Empirical and Model-Based Replication Effect Sizes for Schizophrenia. Mean replication *z*-scores for schizophrenia (SCZ) SNPs from nonparametric estimates (blue curves) and from mixture model based fit (green curves) for varying proportions of discovery and replication sample sizes. *Top row*: Left to right, 10%, 20%, and 30% in training sample. *Middle row*: Left to right, 40%, 50%, and 60% in training sample. *Bottom row*: Left to right, 70%, 80%, and 90% in training sample.
(EPS)

S2 FigExpected posterior effect sizes for normal mixture model.Expected posterior effect sizes for normal mixture model for different parameter values. Note, when $\sigma_{1}^{1}=0$, the line through the origin is flat (has no positive slope). Black line denotes replication effect sizes for the same settings in each plot.(TIF)

S3 Fig Simulation Study 1. Simulated empirical(red) and predicted(blue, using the scale-mixture normal model) conditional mean were shown in the first column. The second column shows simulated empirical(red) and predicted(blue, using the scale-mixture normal model) conditional variance. The third column shows boxplots of parameter estimates across all 50 iterations of the simulation study. The parameter values were set by expanding a grid with *π*_1_ = {0.01, 0.02, 0.05}, *σ*_1_ = {0.0, 0.001, 0.01} and *σ*_2_ = {0.01, 0.05, 0.1}.
(EPS)

S4 Fig Simulation Study 2. Simulated empirical(red) and predicted(blue, using the scale-mixture normal model) conditional mean were shown in the first column. The second column shows simulated empirical(red) and predicted(blue, using the scale-mixture normal model) conditional variance. The third column shows boxplots of parameter estimates across all 50 iterations of the simulation study. The parameter values were set by expanding a grid with *π*_1_ = {0.01, 0.02, 0.05}, *σ*_1_ = {0.0, 0.001, 0.01} and *σ*_2_ = {0.01, 0.05, 0.1}.
(EPS)

S5 Fig Simulation Study 3. Simulated empirical(red) and predicted(blue, using the scale-mixture normal model) conditional mean were shown in the first column. The second column shows simulated empirical(red) and predicted(blue, using the scale-mixture normal model) conditional variance. The third column shows boxplots of parameter estimates across all 50 iterations of the simulation study. The parameter values were set by expanding a grid with *π*_1_ = {0.01, 0.02, 0.05}, *σ*_1_ = {0.0, 0.001, 0.01} and *σ*_2_ = {0.01, 0.05, 0.1}.
(EPS)

S6 Fig Simulation Study 4. Simulated empirical(red) and predicted(blue, using the scale-mixture normal model) conditional mean were shown in the first column. The second column shows simulated empirical(red) and predicted(blue, using the scale-mixture normal model) conditional variance. The third column shows boxplots of parameter estimates across all 50 iterations of the simulation study. The parameter values were set by expanding a grid with *π*_1_ = {0.01, 0.02, 0.05}, *σ*_1_ = {0.0, 0.001, 0.01} and *σ*_2_ = {0.01, 0.05, 0.1}.
(EPS)

S7 Fig Simulation Study 5. Simulated empirical(red) and predicted(blue, using the scale-mixture normal model) conditional mean were shown in the first column. The second column shows simulated empirical(red) and predicted(blue, using the scale-mixture normal model) conditional variance. The third column shows boxplots of parameter estimates across all 50 iterations of the simulation study. The parameter values were set by expanding a grid with *π*_1_ = {0.01, 0.02, 0.05}, *σ*_1_ = {0.0, 0.001, 0.01} and *σ*_2_ = {0.01, 0.05, 0.1}.
(EPS)
