# Supplementary material for: An Empirical Bayes Mixture Model for Effect Size Distributions in Genome-Wide Association Studies
Source: PLoS Genet. 2015 Dec 29;11(12):e1005717. doi: 10.1371/journal.pgen.1005717 (PMC5456456; doi:10.1371/journal.pgen.1005717)
Supplement: S1 Text — Sections 1–2 provide statistical derivations of the effects of linkage disqequilibrium (LD) on massively univariate regression estimates from GWAS. Section 3 outlines how posterior effect size estimates from the mixture model could be used in polygenic risk score estimation. Section 4 provides details and results from Simulation Studies, as described in the main text. Section 5 gives the authorship list for the Psychiatric Genetics Consortium Schizophrenia Working Group. (PDF) [file pgen.1005717.s001.pdf]

Contents

1 Generative Model **2**

2 Regression Estimates **3**

3 Polygenic Risk Prediction **4**

4 Simulation Studies **4**

4.1 Simulation 1 4

4.2 Simulation 2 5

4.3 Simulation 3 5

4.4 Simulation 4 6

4.5 Simulation 5 6

5 Schizophrenia Working Group of the Psychiatric Genomics Consortium **7**

1 Generative Model

In this section we describe a simple multivariate, additive *generative* (causal) model for the genotype-phenotype relationship. While this model is almost certainly a simplification of reality (e.g., ignoring dominance and epistasis), similar models form the basis for many discussions of effect size distributions in the literature (see, e.g., [1, 2]). For the jth subject, $j = 1, \dots, n$, we have data $\{\boldsymbol{x}_j, y_j\}$, where $\boldsymbol{x}_j$ is the vector of mean-centered allele counts from N assayed bi-allelic loci and y_j is a mean-centered quantitative response variable. We treat the dichotomous outcome case below. Let

$$\boldsymbol{X} = \begin{pmatrix} \boldsymbol{x}_1^T \\ \vdots \\ \boldsymbol{x}_n^T \end{pmatrix} = (\boldsymbol{v}_1, \dots, \boldsymbol{v}_N)$$

denote the $n \times N$ matrix of mean-centered allele counts. We assume a simple additive generative model

$$\boldsymbol{Y}^* = \boldsymbol{X}\boldsymbol{\beta} + \boldsymbol{\epsilon} \tag{1}$$

where $\boldsymbol{\beta}$ is an N-dimensional vector of causal (per-allele) effects and $\boldsymbol{\epsilon}$ is an n-dimensional vector containing additive environmental and measurement error effects. If the phenotype is continuous, then the observed $\boldsymbol{Y} = \boldsymbol{Y}^*$. If the phenotype is dichotomous, then $y_j = I_{y_j^* \geq 0}$, where $I_{y_j^* \geq 0} = 1$ if $y_j^* \geq 0$ and zero otherwise. We make the following assumptions:

(A1) Causal loci are a subset of tagged loci.

(A2) The $\boldsymbol{x}_i$ are in Hardy-Weinberg Equilibrium (HWE).

(A3) $\epsilon \sim (\boldsymbol{0}, \sigma_\epsilon^2 \boldsymbol{I})$, for variance $\sigma_\epsilon^2 > 0$ and $n \times n$ identity matrix $\boldsymbol{I}$.

(A4) $\boldsymbol{X} \perp\!\!\!\perp \boldsymbol{\epsilon}$.

(A5) $(\boldsymbol{X}, \boldsymbol{\epsilon}) \perp\!\!\!\perp \boldsymbol{\beta}$ ("Genes and environment independent of per-allele effects").

Assumption (A1) is not necessary for the derivation but simplifies the description of the model. Here, $(\boldsymbol{\mu}, \boldsymbol{\Sigma})$ denotes a multivariate normal distribution with mean $\boldsymbol{\mu}$ and positive definite covariance matrix $\boldsymbol{\Sigma}$. If the phenotype is continuous, we typically make the additional assumption that $\boldsymbol{\epsilon}$ is multivariate normal. If the phenotype is dichotomous, assuming that the ϵ_i are independent with the logistic distribution leads to a logistic regression model framework. Under Assumption (A2), the x_{ij} are random draws from $X_i \sim \text{Bin}(2, p_i) - 2p_i$, so that $\text{E}\{X_i\} = 0$ and $\text{Var}\{X_i\} = 2p_i(1 - p_i)$, where p_i is the population proportion of

reference alleles for the ith SNP, and we neglect uncertainty arising from estimation of p_i. We also assume that the marginal density g of the causal effects β_i is symmetric around zero with finite first and second moments, so that $\mathrm{E}\{\beta_i\} = 0$ and $\sigma_\beta^2 \equiv \mathrm{E}\{\beta_i^2\} = \mathrm{Var}\{\beta_i\}$.

2 Regression Estimates

Here, for ease of presentation we focus the development on genome-wide analyses of quantitative traits. We assume the association of the ith SNP with the quantitative trait is assessed via univariate linear regression. In the absence of covariates, the least squares estimates can be expressed as

$$\begin{aligned}\widehat{b}_i &= \frac{\boldsymbol{v}_i^T \boldsymbol{y}}{\boldsymbol{v}_i^T \boldsymbol{v}_i}, \\ &= \sum_{i'=1}^{N} \frac{\boldsymbol{v}_i^T \boldsymbol{v}_{i'}}{\boldsymbol{v}_i^T \boldsymbol{v}_i} \beta_{i'} + \frac{\boldsymbol{v}_i^T \boldsymbol{\epsilon}}{\boldsymbol{v}_i^T \boldsymbol{v}_i} \\ &= \beta_i + \sum_{i' \neq i} \widehat{\xi}_{ii'} \beta_{i'} + \frac{\boldsymbol{v}_i^T \boldsymbol{\epsilon}}{\boldsymbol{v}_i^T \boldsymbol{v}_i}, \quad i = 1, \ldots, N, \qquad (2)\end{aligned}$$

where $\widehat{\xi}_{ii'} = \boldsymbol{v}_i^T \boldsymbol{v}_{i'} / \boldsymbol{v}_i^T \boldsymbol{v}_i = \widehat{\lambda}_{ii'} / \widehat{\lambda}_{ii}$, with $\widehat{\lambda}_{ii'} = \boldsymbol{v}_i^T \boldsymbol{v}_{i'} / n$, for $i, i' = 1, \ldots, N$. The $\widehat{\lambda}_{ii'}$ are estimates of the covariance between the ith and i'th SNPs, with

$$\lambda_{ii'} \equiv \mathrm{E}_X \left\{ \widehat{\lambda}_{ii'} \right\} = \sqrt{2p_i(1-p_i)} \sqrt{2p_{i'}(1-p_{i'})} \rho_{ii'},$$

where $\rho_{ii'}$ is the correlation between the ith and i'th SNPs. Let $\xi_{ii'} = \mathrm{E}_X\{\widehat{\xi}_{ii'}\}$, the regression coefficient of the i'th SNP on the ith SNP, so that

$$\widehat{\xi}_{ii'} \simeq \xi_{ii'} = \frac{\sqrt{p_{i'}(1-p_{i'})}}{\sqrt{p_i(1-p_i)}} \rho_{ii'}.$$

The symbol "$\simeq$" denotes asymptotic equality as $n \to \infty$. Thus,

$$b_i \equiv \mathrm{E}\{\widehat{b}_i\} = \beta_i + \sum_{i' \neq i} \xi_{ii'} \beta_{i'}, \quad i = 1, \ldots, N, \qquad (3)$$

and hence the bias in estimation of β_i from univariate regression estimates $\widehat{b}_i$ depends on the distribution of causal effects $\beta_{i'}$, as well as the distribution of $\xi_{ii'}$, for $i' = 1, \ldots, N$. In particular, non-null ("large") effects b_i have (relatively) large values of $\sum_{i'=1}^{N} \xi_{ii'} \beta_{i'}$, even if β_i is itself zero.

3 Polygenic Risk Prediction

Posterior expectations $\mathrm{E}\{\delta_i \mid Z_i = z_i\}, i = 1, \ldots, N$, can be used to compute polygenic risk scores, defined below. Suppose the phenotype for the jth subject is dichotomous, $Y_j \in \{0, 1\}$, where 0 indicates a control and 1 a patient. Let $\gamma_i = p_{1i} - p_{0i}, i = 1, \ldots, N$, where p_{1i} is the proportion of reference alleles in the patients and p_{0i} in the controls. If there are an equal number of patients and controls in the sample, $p_i = (p_{0i} + p_{1i})/2$, and hence

$$\mathrm{E}\{U_{ij}\} = \begin{cases} -\dfrac{\gamma_i}{\sigma_i}, & \text{if subject } j \text{ is a control,} \\ \dfrac{\gamma_i}{\sigma_i}, & \text{if subject } j \text{ is a patient.} \end{cases}$$

Define the *polygenic risk score* for the jth subject as $S_j = \sum_{i=1}^{N} \delta_i U_{ij}$. The S_j are approximately distributed as $\mathrm{N}(\pm\boldsymbol{\delta}'\boldsymbol{\gamma}, \tau^2)$, where $\boldsymbol{\delta} = (\delta_1, \ldots, \delta_N)'$, $\boldsymbol{\gamma} = (\gamma_1, \ldots, \gamma_N)'$, $\tau^2 \approx \|\boldsymbol{\delta}\|^2$, and $\pm\boldsymbol{\delta}'\boldsymbol{\gamma}$ is positive if the subject is a patient and negative if the subject is a control. Subject j in the replication sample is predicted to be a control if $\widehat{S}_j < 0$ and a patient if $\widehat{S}_j > 0$.

4 Simulation Studies

4.1 Simulation 1

We evaluated the performance of the estimation method by simulating summary statistics (z-scores) of 30 sub-studies with varying effective sample sizes. One million diallelic SNPs with minor allele frequencies (MAF) sampled $U(0.005, 0.5)$ were simulated. Twenty-seven scenarios were constructed from all possible combinations of the following parameter levels: $\pi_1 = \{0.01, 0.02, 0.05\}$, $\sigma_1 = \{0, 0.001, 0.01\}$ and $\sigma_2 = \{0.01, 0.05, 0.1\}$, each of which were repeated 50 times with different random seeds. We set $\sigma_0 = 1$ for all simulations. The z-scores for each sub-study were sampled from the scale-mixture of two normals distribution as in Eq. (11) in the main text. The constructed sub-studies were randomly split in half 100 times and the quadratic estimating equation method was applied to get the estimates of the parameters $\boldsymbol{\theta} = \{\pi_1, \sigma_0, , \sigma_1, , \sigma_2\}$. To study the effect of departures from the model assumptions (a)-(c) on parameter estimates, we included sub-study stratification by varying MAF among sub-studies (adding in independent $U(0.01, 0.10)$ random variables to each $p_{k,i}$ where $k = 1, \ldots 30$ indicates sub-study and $i = 1, \ldots, N$ denotes the SNP number. Assumptions (a)-(c) all have the effect of keeping the effect sizes $\delta_{k,i} = \delta_i$ for all sub-studies. Any random departures from these assumptions imply $\delta_{k,i} \neq \bar{\delta}_i$, where $\bar{\delta}_i$ is the mean effect size across sub-studies for the ith SNP. As we note in the main text, assumptions (a)-(c) are standard assumptions for fixed effects meta-analyses (which were applied to compute mea-analysis p-values in both the CD

and SCZ papers [3, 4]. If (a)-(c) are not valid, a random effects meta-analysis can be applied instead [5].

S3 Fig shows the results for the 27 simulations for the different settings of π_1,σ_1, and σ_2. Empirical and model based posterior means and variances are quite close, and the parameter estimates from the quadratic equations are unbiased with reasonably small levels of variability across simulations. This demonstrates that the algorithm is able to recover the ground truth when independent SNPs are to fit the algorithm.

4.2 Simulation 2

We also studied if the impact of linkage disequilibrium (LD) between SNPs, and if this could be the source of the observed non-zero small effect variance σ_1, thus accounting for the observed non-zero slope around the origin. The z-scores of SNPs in a LD block were sampled from a multivariate normal distribution for each sub-study. The size of LD block, in terms of number of SNPs, were sampled from $U(50, 100)$. We assume that the SNP in the middle of the LD block has the largest effect, and the effect decays both directions. To model this decay, we constructed a exponential decay with starting value=1, and stop value= 0.7. Then, the σ_2 value of the middle SNPs were multiplied by the decay parameter. The covariance matrix of the multivariate normal was then constructed by taking the outer product of the computed decaying σ_2.

S4 fig shows the effects of randomly jittering MAF across sub-studies by adding large independent differences to MAF at each SNP. The settings for this simulation were chosen to be close to the estimates from the CD study. The effects of large random differences across sub-studies is to slightly raise estimates of σ_0 but lowering estimates of σ_1 and σ_2. The non-null proportion is largely un-affected, actually being slightly closer to the true values in the jittered MAF simulations. The level of variation in MAFs across sub-studies is quite large, a scenario under which fixed-effect meta-analyses are inappropriate. Simulations with smaller random variations (not shown here) show much reduced effects on parameter estimates.

4.3 Simulation 3

S5 Fig shows the impact of LD, again using parameter values close to that of the CD example, except with $\sigma_1 - 0$. The first row shows the simulations from. The posterior expectations, variances, and parameter estimates are very close to the ground truth with no LD, as in the first set of simulations. In the second row, the effects of LD are apparent: the "small" effects are now non-zero, which is apparent both from the non-zero slope of the posterior expectations and in the median estimate of $\widehat{\sigma}_1 = 0.001$. Also, the median estimate of the non-null proportion is much higher than the non-null proportion of the generative effects. The fitting algorithm still captures the distribution of effects on the z-scores (as can be seen by the very close fits of the posterior means and variances in the first and second columns), but the interpretation of

these effects is different, i.e., the interpretation is in terms of the posterior expectation and variances of the (massively univariate) regression estimates $\widehat{b}_i$ rather than the causal effects β_i, $i = 1, \ldots, N$.

4.4 Simulation 4

S6 Fig repeats the same simulations as for bf S4 Fig, but with parameter setting that mirror the estimated from the schizophrenia real data application. These result in qualitatively similar results.

4.5 Simulation 5

S7 Fig repeats the same simulations as in **S5 Fig**, but with parameter setting that mirror the estimated from the schizophrenia real data application. These also result in qualitatively similar results; however, as can be seen in the second and third rows of **S7 Fig**, the impact of LD on inflating pi_1 and in creating small but pervasive replicating effects is more dramatically apparent.

5 Schizophrenia Working Group of the Psychiatric Genomics Consortium

Stephan Ripke[1,2], Benjamin M. Neale[1,2,3,4], Aiden Corvin[5], James T. R. Walters[6], Kai-How Farh[1], Peter A. Holmans[6,7], Phil Lee[1,2,4], Brendan Bulik-Sullivan[1,2], David A. Collier[8,9], Hailiang Huang[1,3], Tune H. Pers[3,10,11], Ingrid Agartz[12,13,14], Esben Agerbo[15,16,17], Margot Albus[18], Madeline Alexander[19], Farooq Amin[20,21], Silviu A. Bacanu[22], Martin Begemann[23], Richard A. Belliveau Jr[2], Judit Bene[24,25], Sarah E. Bergen[2,26], Elizabeth Bevilacqua[2], Tim B. Bigdeli[22], Donald W. Black[27], Richard Bruggeman[28], Nancy G. Buccola[29], Randy L. Buckner[30,31,32], William Byerley[33], Wiepke Cahn[34], Guiqing Cai[35,36], Dominique Campion[37], Rita M. Cantor[38], Vaughan J. Carr[39,40], Noa Carrera[6], Stanley V. Catts[39,41], Kimberly D. Chambert[2], Raymond C. K. Chan[42], Ronald Y. L. Chen[43], Eric Y. H. Chen[43,44], Wei Cheng[45], Eric F. C. Cheung[46], Siow Ann Chong[47], C. Robert Cloninger[48], David Cohen[49], Nadine Cohen50, Paul Cormican[5], Nick Craddock[6,7], James J. Crowley[51], David Curtis[52,53], Michael Davidson[54], Kenneth L. Davis[36], Franziska Degenhardt[55,56], Jurgen Del Favero57, Ditte Demontis[17,58,59], Dimitris Dikeos[60], Timothy Dinan[61], Srdjan Djurovic[14,62], Gary Donohoe[5,63], Elodie Drapeau[36], Jubao Duan[64,65], Frank Dudbridge[66], Naser Durmishi[67], Peter Eichhammer[68], Johan Eriksson[69,70,71], Valentina Escott-Price[6], Laurent Essioux[72], Ayman H. Fanous[73,74,75,76], Martilias S. Farrell[51], Josef Frank[77], Lude Franke[78], Robert Freedman[79], Nelson B. Freimer[80], Marion Friedl[81], Joseph I. Friedman[36], Menachem Fromer[1,2,4,82], Giulio Genovese[2], Lyudmila Georgieva[6], Ina Giegling[81,83], Paola Giusti-Rodrguez[51], Stephanie Godard[84], Jacqueline I. Goldstein[1,3], Vera Golimbet[85], Srihari Gopal[86], Jacob Gratten[87], Lieuwe de Haan[88], Christian Hammer[23], Marian L. Hamshere[6], Mark Hansen[89], Thomas Hansen[17,90], Vahram Haroutunian[36,91,92], Annette M. Hartmann[81], Frans A. Henskens[39,93,94], Stefan Herms[55,56,95], Joel N. Hirschhorn[3,11,96], Per Hoffmann[55,56,95], Andrea Hofman[55,56], Mads V. Hollegaard[97], David M. Hougaard[97], Masashi Ikeda98, Inge Joa[99], Antonio Julia [100], Rene S. Kahn[34], Luba Kalaydjieva[101,102], Sena Karachanak-Yankova[103], Juha Karjalainen[78], David Kavanagh[6], Matthew C. Keller[104], James L. Kennedy[105,106,107], Andrey Khrunin[108], Yunjung Kim51, Janis Klovins[109], James A. Knowles[110], Bettina Konte[81], Vaidutis Kucinskas[111], Zita Ausrele Kucinskiene[111], Hana Kuzelova-Ptackova[112], Anna K. Kahler[26], Claudine Laurent[19,113], Jimmy Lee Chee Keong[47,114], S. Hong Lee[87], Sophie E. Legge6, Bernard Lerer[115], Miaoxin Li[43,44,116], Tao Li[117], Kung-Yee Liang[118], Jeffrey Lieberman[119], Svetlana Limborska[108], Carmel M. Loughland[39,120], Jan Lubinski[121], Jouko Lonnqvist[122], Milan Macek Jr[112], Patrik K. E. Magnusson[26], Brion S. Maher[123], Wolfgang Maier[124], Jacques Mallet[125], Sara Marsal[100], Manuel Mattheisen[17,58,59,126], Morten Mattingsdal[14,127], Robert W. McCarley[128,129], Colm McDonald[130], Andrew M. McIntosh[131,132], Sandra Meier[77], Carin J. Meijer[88], Bela Melegh[24,25], Ingrid Melle[14,33], Raquelle I. Mesholam-Gately[128,134], Andres Metspalu[135], Patricia T. Michie[39,136], Lili Milani1[35], Vihra Milanova[137], Younes Mokrab[8], Derek W.

Morris[5,63], Ole Mors[17,58,138], Kieran C. Murphy[139], Robin M. Murray[140], Inez Myin-Germeys[141], Bertram Muller-Myhsok[142,143,144], Mari Nelis[135], Igor Nenadic[145], Deborah A. Nertney[146], Gerald Nestadt[147], Kristin K. Nicodemus[148], Liene Nikitina-Zake[109], Laura Nisenbaum[149], Annelie Nordin[150], Eadbhard OCallaghan[151], Colm ODushlaine[2], F. Anthony ONeill1[52], Sang-Yun Oh1[53], Ann Olincy[79], Line Olsen[17,90], Jim Van Os[141,154], Psychosis Endophenotypes International Consortium[155], Christos Pantelis[39,156], George N. Papadimitriou[60], Sergi Papiol[23], Elena Parkhomenko[36], Michele T. Pato[110], Tiina Paunio[157,158], Milica Pejovic-Milovancevic[159], Diana O. Perkins160, Olli Pietilainen[158,161], Jonathan Pimm[53], Andrew J. Pocklington[6], John Powell[140], Alkes Price3,[162], Ann E. Pulver[147], Shaun M. Purcel[l82], Digby Quested[163], Henrik B. Rasmussen[17,90], Abraham Reichenberg[36], Mark A. Reimers[164], Alexander L. Richards[6], Joshua L. Roffman[30,32], Panos Roussos[82,165], Douglas M. Ruderfer[6,82], Veikko Salomaa[71], Alan R. Sanders[64,65], Ulrich Schall[39,120], Christian R. Schubert[166], Thomas G. Schulze[77,167], Sibylle G. Schwab[168], Edward M. Scolnick[2], Rodney J. Scott[39,169,170], Larry J. Seidman[128,134], Jianxin Shi[171], Engilbert Sigurdsson[172], Teimuraz Silagadze[173], Jeremy M. Silverman[36,174], Kang Sim[47], Petr Slominsky[108], Jordan W. Smoller[2,4], Hon-Cheong So[43], Chris C. A. Spencer[175], Eli A. Stahl[3,82], Hreinn Stefansson[176], Stacy Steinberg[176], Elisabeth Stogmann[177], Richard E. Straub[178], Eric Strengman[179,34], Jana Strohmaier[77], T. Scott Stroup[119], Mythily Subramaniam[47], Jaana Suvisaari[122], Dragan M. Svrakic[48], Jin P. Szatkiewicz[51], Erik Soderman[12], Srinivas Thirumalai[180], Draga Toncheva[103], Sarah Tosato[181], Juha Veijola[182,183], John Waddington[184], Dermot Walsh[185], Dai Wang[86], Qiang Wang[117], Bradley T. Webb[22], Mark Weiser[54], Dieter B. Wildenauer[186], Nigel M. Williams[6], Stephanie Williams[51], Stephanie H. Witt[77], Aaron R. Wolen[164], Emily H. M. Wong[43], Brandon K. Wormley[22], Hualin Simon Xi[187], Clement C. Zai[105,106], Xuebin Zheng[188], Fritz Zimprich[177], Naomi R. Wray[87], Kari Stefansson[176], Peter M. Visscher[87], Wellcome Trust Case-Control Consortium 2[189], Rolf Adolfsson[150], Ole A. Andreassen[14,133], Douglas H. R. Blackwood[132], Elvira Bramon[190], Joseph D. Buxbaum[35,36,91,191], Anders D. Brglum[17,58,59,138], Sven Cichon[55,56,95,192], Ariel Darvasi[193], Enrico Domenici[194], Hannelore Ehrenreich[23], Tonu Esko[3,11,96,135], Pablo V. Gejman[64,65], Michael Gill[5], Hugh Gurling[53], Christina M. Hultman[26], Nakao Iwata[98], Assen V. Jablensky[39,102,186,195], Erik G. Jonsson[12,14], Kenneth S. Kendler[196], George Kirov[6], Jo Knight[105,106,107], Todd Lencz[197,198,199], Douglas F. Levinson[19], Qingqin S. Li[86], Jianjun Liu[188,200], Anil K. Malhotra[197,198,199], Steven A. McCarroll[2,96], Andrew McQuillin[53], Jennifer L. Moran[2], Preben B. Mortensen[15,16,17], Bryan J. Mowry[87,201], Markus M. Nothen[55,56], Roel A. Ophoff[38,80,34], Michael J. Owen[6,7], Aarno Palotie[2,4,161], Carlos N. Pato[110], Tracey L. Petryshen[2,128,202], Danielle Posthuma[203,204,205], Marcella Rietschel[77], Brien P. Riley[196], Dan Rujescu[81,83], Pak C. Sham[43,44,116], Pamela Sklar[82,91,165], David St Clair[206], Daniel R. Weinberger[178,207], Jens R. Wendland[166], Thomas Werge[17,90,208], Mark J. Daly[1,2,3], Patrick F. Sullivan[26,51,160] & Michael C. ODonovan[6,7]

[1]Analytic and Translational Genetics Unit, Massachusetts General Hospital, Boston, Massachusetts

02114, USA. [2]Stanley Center for Psychiatric Research, Broad Institute of MIT and Harvard, Cambridge, Massachusetts 02142, USA. [3]Medical and Population Genetics Program, Broad Institute of MIT and Harvard, Cambridge, Massachusetts 02142, USA. [4]Psychiatric and Neurodevelopmental Genetics Unit, Massachusetts General Hospital, Boston, Massachusetts 02114, USA. [5]Neuropsychiatric Genetics Research Group, Department of Psychiatry, Trinity College Dublin, Dublin 8, Ireland. [6]MRC Centre for Neuropsychiatric Genetics and Genomics, Institute of Psychological Medicine and Clinical Neurosciences, School ofMedicine, Cardiff University, Cardiff CF244HQ, UK. [7]NationalCentre forMentalHealth, CardiffUniversity, Cardiff CF244HQ,UK. [8]Eli Lilly and Company Limited, Erl Wood Manor, Sunninghill Road, Windlesham, Surrey GU20 6PH, UK. [9]Social, Genetic and Developmental Psychiatry Centre, Institute of Psychiatry, Kings College London, London SE5 8AF,UK. [10]Center for Biological Sequence Analysis, Department of Systems Biology, Technical University of Denmark, DK-2800, Denmark. [11]Division of Endocrinology and Center for Basic and Translational Obesity Research, Boston Childrens Hospital, Boston,Massachusetts02115,USA. [12]Department of Clinical Neuroscience, Psychiatry Section, Karolinska Institutet, SE-17176 Stockholm, Sweden. [13]Department of Psychiatry, Diakonhjemmet Hospital, 0319 Oslo, Norway. [14]NORMENT, KG Jebsen Centre for Psychosis Research, Institute of Clinical Medicine, University of Oslo, 0424 Oslo, Norway. [15]Centre for Integrative Register-based Research, CIRRAU, Aarhus University, DK-8210 Aarhus, Denmark. [16]National Centre for Register-basedResearch, AarhusUniversity,DK-8210Aarhus,Denmark. [17]The Lundbeck Foundation Initiative for Integrative Psychiatric Research, iPSYCH, Denmark. [18]State Mental Hospital, 85540 Haar, Germany. [19]Department of Psychiatry and Behavioral Sciences, Stanford University, Stanford, California 94305, USA. [20]Department of Psychiatry and Behavioral Sciences, Atlanta Veterans Affairs Medical Center, Atlanta, Georgia 30033, USA. [21]Department of Psychiatry and Behavioral Sciences, Emory University, Atlanta, Georgia30322,USA. [22]Virginia Institute for Psychiatric and Behavioral Genetics, Department of Psychiatry, Virginia Commonwealth University, Richmond, Virginia 23298, USA. [23]Clinical Neuroscience, Max Planck Institute of Experimental Medicine, Gottingen 37075, Germany. [24]Department of Medical Genetics, University of Pecs, Pecs H-7624, Hungary. [25]Szentagothai Research Center, University of Pecs, Pecs H-7624, Hungary. [26]Department of Medical Epidemiology and Biostatistics, Karolinska Institutet, Stockholm SE-17177, Sweden. [27]Department of Psychiatry, University of Iowa Carver College of Medicine, Iowa City, Iowa 52242, USA. [28]University Medical Center Groningen, Department of Psychiatry, University of Groningen NL-9700 RB, The Netherlands. [29]School of Nursing, Louisiana StateUniversity Health Sciences Center,New Orleans, Louisiana 70112, USA. [30]Athinoula A. Martinos Center, Massachusetts General Hospital, Boston, Massachusetts 02129, USA. [31]Center for Brain Science, Harvard University, Cambridge, Massachusetts 02138, USA. [32]Department of Psychiatry, Massachusetts General Hospital, Boston, Massachusetts 02114, USA. [33]Department of Psychiatry, University of California at San Francisco, San

Francisco, California 94143,USA. [34]University Medical Center Utrecht, Department of Psychiatry, Rudolf Magnus Institute of Neuroscience, 3584 Utrecht, The Netherlands. [35]Department of Human Genetics, Icahn School of Medicine at Mount Sinai, New York, New York 10029, USA. [36]Department of Psychiatry, IcahnSchool ofMedicine atMount Sinai,NewYork,NewYork 10029, USA. [37]Centre Hospitalier du Rouvray and INSERM U1079 Faculty of Medicine, 76301 Rouen, France. [38]Department of Human Genetics, David Geffen School of Medicine, University of California, Los Angeles, California 90095, USA. [39]Schizophrenia Research Institute,SydneyNSW2010,Australia. [40]School of Psychiatry,University ofNew South Wales, Sydney NSW 2031, Australia. [41]Royal Brisbane and Womens Hospital, University of Queensland, Brisbane, St Lucia QLD 4072, Australia. [42I]nstitute of Psychology, Chinese Academy of Science, Beijing 100101, China. [43]Department of Psychiatry, Li Ka Shing Faculty of Medicine, The University of Hong Kong, Hong Kong,China. [44]State Key Laboratory for Brain and Cognitive Sciences, Li Ka Shing Faculty of Medicine, The University of Hong Kong, Hong Kong, China. [45]Department of Computer Science, University of North Carolina, Chapel Hill, North Carolina 27514, USA. [46]Castle Peak Hospital, Hong Kong, China. [47]Institute of Mental Health, Singapore 539747, Singapore. [48]Department of Psychiatry,WashingtonUniversity, St. Louis,Missouri 63110, USA. [49]Department of Child and Adolescent Psychiatry, Assistance Publique Hopitaux de Paris, Pierre and Marie Curie Faculty of Medicine and Institute for Intelligent Systems and Robotics, Paris 75013, France. [50]Blue Note Biosciences, Princeton, New Jersey 08540, USA [51]Department of Genetics, University of North Carolina, Chapel Hill, North Carolina 27599-7264, USA. [52]Department of Psychological Medicine, Queen Mary University of London, London E1 1BB, UK. [53]Molecular Psychiatry Laboratory, Division of Psychiatry, University College London, LondonWC1E6JJ,UK. [54]ShebaMedicalCenter, Tel Hashomer 52621, Israel. [55]Department of Genomics, Life and Brain Center, D-53127 Bonn, Germany. [56]Institute of Human Genetics, University of Bonn, D-53127 Bonn, Germany. [57]Applied Molecular Genomics Unit, VIB Department of Molecular Genetics, University of Antwerp, B-2610 Antwerp, Belgium. [58]Centre for Integrative Sequencing, iSEQ, Aarhus University, DK-8000 Aarhus C, Denmark. [59]Department of Biomedicine, Aarhus University, DK-8000 Aarhus C, Denmark. [60]First Department of Psychiatry, University of Athens Medical School, Athens 11528, Greece. [61]Department of Psychiatry, University College Cork, Co. Cork, Ireland. [62]Department of Medical Genetics, Oslo University Hospital, 0424 Oslo, Norway. [63]Cognitive Genetics and Therapy Group, School of Psychology and Discipline of Biochemistry, National University of Ireland Galway, Co. Galway, Ireland. [64]Department of Psychiatry and Behavioral Neuroscience, University of Chicago, Chicago, Illinois 60637, USA. [65]Department of Psychiatry and Behavioral Sciences, NorthShore University HealthSystem, Evanston, Illinois 60201, USA. [66]Department of Non-Communicable Disease Epidemiology, London School of Hygiene and Tropical Medicine, London WC1E 7HT, UK. [67]Department of Child and Adolescent Psychiatry, University Clinic of Psychiatry, Skopje 1000, Republic of Macedonia.

[68]Department of Psychiatry, University of Regensburg, 93053 Regensburg, Germany. [69]Department of General Practice, Helsinki University Central Hospital, University of Helsinki P.O. Box 20, Tukholmankatu 8 B, FI-00014, Helsinki, Finland [70]Folkhalsan Research Center, Helsinki, Finland, BiomedicumHelsinki 1, Haartmaninkatu 8, FI-00290, Helsinki, Finland. [71]National Institute for Health and Welfare, P.O. Box 30, FI-00271 Helsinki, Finland. [72]Translational Technologies and Bioinformatics, Pharma Research and Early Development, F. Hoffman-La Roche, CH-4070 Basel, Switzerland. [73]Department of Psychiatry, Georgetown University School of Medicine, Washington DC 20057, USA. [74]Department of Psychiatry, Keck School of Medicine of the University of Southern California, Los Angeles, California 90033, USA. [75]Department of Psychiatry, Virginia Commonwealth University School of Medicine, Richmond, Virginia 23298, USA. [76]Mental Health Service Line, Washington VA Medical Center, Washington DC 20422, USA. [77]Department of Genetic Epidemiology in Psychiatry, Central Institute of Mental Health, Medical Faculty Mannheim, University of Heidelberg, Heidelberg , D-68159 Mannheim, Germany. [78]Department of Genetics, University of Groningen, University Medical Centre Groningen, 9700 RB Groningen, The Netherlands. [79]Department of Psychiatry, University of Colorado Denver, Aurora, Colorado 80045, USA. [80]Center for Neurobehavioral Genetics, Semel Institute for Neuroscience and Human Behavior, University of California, Los Angeles, California 90095, USA. [81]Department of Psychiatry, University of Halle, 06112 Halle, Germany. [82]Division of Psychiatric Genomics, Department of Psychiatry, Icahn School of Medicine at Mount Sinai, New York, New York, NewYork 10029,USA. [83]Department of Psychiatry,University ofMunich, 80336,Munich, Germany. [84]Departments of Psychiatry and Human and Molecular Genetics, INSERM, Institut de Myologie, Hopital de la Pitie-Salpetriere, Paris 75013, France. [85]Mental Health Research Centre, Russian Academy of Medical Sciences, 115522 Moscow, Russia. [86]Neuroscience Therapeutic Area, Janssen Research and Development, Raritan, New Jersey 08869, USA. [87]Queensland Brain Institute, The University of Queensland, Brisbane, Queensland, QLD 4072, Australia. [88]Academic Medical Centre University of Amsterdam, Department of Psychiatry, 1105 AZ Amsterdam, The Netherlands. [89]*I*llumina, La Jolla, California, California 92122, USA. [90]Institute of Biological Psychiatry, MentalHealthCentre Sct.Hans,MentalHealthServicesCopenhagen,DK-4000,Denmark. [91]riedman Brain Institute, Icahn School of Medicine at Mount Sinai, New York, New York 10029, USA. [92]J. J. Peters VA Medical Center, Bronx, New York, New York 10468, USA. [93]Priority Research Centre for Health Behaviour, University of Newcastle, Newcastle NSW 2308, Australia. [94]School of Electrical Engineering and Computer Science, University of Newcastle, Newcastle NSW 2308, Australia. [95]Division of Medical Genetics, Department of Biomedicine, University of Basel, Basel CH-4058, Switzerland. [96]Department of Genetics, Harvard Medical School, Boston, Massachusetts, Massachusetts 02115, USA. [97]Section of Neonatal Screening and Hormones, Department of Clinical Biochemistry, Immunology and Genetics, Statens Serum Institut, Copenhagen DK-2300, Denmark. [98]Department of Psychiatry, Fu-

jita Health University School of Medicine, Toyoake, Aichi,470-1192, Japan. [99]Regional Centre for Clinical Research in Psychosis,Department of Psychiatry, Stavanger University Hospital, 4011 Stavanger, Norway. [100]Rheumatology Research Group, Vall dHebron Research Institute, Barcelona 08035, Spain. [101]Centre for Medical Research, The University of Western Australia, Perth WA6009, Australia. [102]The Perkins Institute for Medical Research, The University of Western Australia, Perth WA6009, Australia. [103]Department of Medical Genetics, Medical University, Sofia 1431, Bulgaria. [104]Department of Psychology, University of Colorado Boulder, Boulder, Colorado 80309, USA. [105]Campbell Family Mental Health Research Institute, Centre for Addiction and Mental Health, Toronto, Ontario M5T 1R8, Canada. [106]Department of Psychiatry, University of Toronto, Toronto, Ontario M5T 1R8, Canada. [107]*I*nstitute of Medical Science, University of Toronto, Toronto, Ontario M5S 1A8, Canada. [108]Institute of Molecular Genetics, Russian Academy of Sciences, Moscow 123182, Russia. [109]Latvian Biomedical Research and Study Centre, Riga, LV-1067, Latvia. [110]Department of Psychiatry and Zilkha Neurogenetics Institute, Keck School of Medicine at University of Southern California, Los Angeles, California 90089, USA. [111]Faculty of Medicine, Vilnius University, LT-01513 Vilnius, Lithuania. [112]Department of Biology and Medical Genetics, 2nd Faculty of Medicine and University Hospital Motol, 150 06 Prague, Czech Republic. [113]Department of Child and Adolescent Psychiatry, Pierre and Marie Curie Faculty of Medicine, Paris 75013, France. [114]Duke-NUS Graduate Medical School, Singapore 169857. [115]Department of Psychiatry, Hadassah-Hebrew University Medical Center, Jerusalem 91120, Israel. [116]Centre for Genomic Sciences, The University of Hong Kong, Hong Kong, China. [117]Mental Health Centre and Psychiatric Laboratory, West China Hospital, Sichuan University, Chengdu, 610041 Sichuan, China. [118]Department of Biostatistics, Johns Hopkins University Bloomberg School of Public Health, Baltimore, Maryland 21205, USA. [119]Department of Psychiatry, ColumbiaUniversity, NewYork, New York 10032, USA. [120]Priority Centre for Translational Neuroscience and Mental Health, University of Newcastle, Newcastle NSW2300, Australia. [121]Department of Genetics and Pathology, International Hereditary Cancer Center, Pomeranian Medical University in Szczecin, 70-453 Szczecin, Poland. [122]Department of Mental Health and Substance Abuse Services; National Institute for Health and Welfare, P.O. BOX30, FI-00271 Helsinki, Finland. [123]Department of Mental Health, Bloomberg School of Public Health, Johns Hopkins University, Baltimore, Maryland 21205, USA. [124]Department of Psychiatry, University of Bonn, D-53127 Bonn, Germany. [125]Centre National de la Recherche Scientifique, Laboratoire de Genetique Moleculaire de la Neurotransmission et des Processus Neurodegeneratifs, Hopital de la Pitie Salpetrie‘re, 75013 Paris, France. [126]Department of Genomics Mathematics, University of Bonn, D-53127 Bonn, Germany. [127]Research Unit, Srlandet Hospital, 4604 Kristiansand, Norway. [128]Department of Psychiatry, Harvard Medical School, Boston, Massachusetts 02115, USA. [129]VA Boston Health Care System, Brockton, Massachusetts 02301, USA. [130]Department of Psychiatry, National University of Ireland Galway, Co. Galway,

Ireland. [131]Centre for Cognitive Ageing and Cognitive Epidemiology, University of Edinburgh, Edinburgh EH16 4SB, UK. [132]Division of Psychiatry, University of Edinburgh, Edinburgh EH16 4SB, UK. [133]Division of Mental Health and Addiction, Oslo University Hospital, 0424 Oslo, Norway. [134]Massachusetts Mental Health Center Public Psychiatry Division of the Beth Israel Deaconess Medical Center, Boston, Massachusetts 02114, USA. [135]Estonian Genome Center, University of Tartu, Tartu 50090, Estonia. [136]School of Psychology, University of Newcastle, NewcastleNSW2308, Australia. [137]First Psychiatric Clinic,Medical University, Sofia 1431, Bulgaria. [138]Department P, Aarhus University Hospital, DK-8240 Risskov, Denmark. [139]Department of Psychiatry, Royal College of Surgeons in Ireland, Dublin 2, Ireland. [140]Kings College London, London SE5 8AF, UK. [141]MaastrichtUniversity Medical Centre, South Limburg Mental Health Research and Teaching Network, EURON, 6229 HX Maastricht, The Netherlands. [142]Institute of Translational Medicine, University of Liverpool, Liverpool L69 3BX, UK. [143]Max Planck Institute of Psychiatry, 80336 Munich, Germany. [144]Munich Cluster for SystemsNeurology (SyNergy),80336Munich,Germany. [145]Department of Psychiatry and Psychotherapy, Jena University Hospital, 07743 Jena, Germany. [146]Department of Psychiatry, Queensland Brain Institute and Queensland Centre for Mental Health Research, University of Queensland, Brisbane, Queensland, St Lucia QLD 4072, Australia. [147]Department of Psychiatry and Behavioral Sciences, Johns Hopkins University School ofMedicine, Baltimore, Maryland 21205, USA. [148]Department of Psychiatry, Trinity College Dublin, Dublin 2, Ireland. [149]Eli Lilly and Company, Lilly Corporate Center, Indianapolis, 46285 Indiana, USA. [150]Department of Clinical Sciences, Psychiatry, Umea University, SE-901 87 Umea, Sweden. [151]DETECT Early Intervention Service for Psychosis, Blackrock, Co. Dublin, Ireland. [152]Centre for Public Health, Institute of Clinical Sciences, Queens University Belfast, Belfast BT12 6AB, UK. [153]Lawrence Berkeley National Laboratory, University of California at Berkeley, Berkeley, California 94720, USA. [154]Institute of Psychiatry, Kings College London, London SE5 8AF, UK. [155] [156]Melbourne Neuropsychiatry Centre, University of Melbourne & Melbourne Health, Melbourne, Vic 3053, Australia. [157]Department of Psychiatry, University of Helsinki, P.O. Box 590, FI-00029 HUS, Helsinki, Finland. [158]Public Health Genomics Unit, National Institute for Health and Welfare, P.O. BOX 30, FI-00271 Helsinki, Finland [159]Medical Faculty, University of Belgrade, 11000 Belgrade, Serbia. [160]Department of Psychiatry, University of North Carolina, Chapel Hill, North Carolina 27599-7160, USA. [161]*I*nstitute for Molecular Medicine Finland, FIMM, University of Helsinki, P.O. Box 20FI-00014, Helsinki, Finland [162]Department of Epidemiology, Harvard School of Public Health, Boston, Massachusetts 02115, USA. [163]Department of Psychiatry, University of Oxford, Oxford, OX3 7JX, UK. [164]Virginia Institute for Psychiatric and Behavioral Genetics, Virginia Commonwealth University, Richmond, Virginia 23298, USA. [165]Institute for Multiscale Biology, Icahn School of Medicine at Mount Sinai, New York, New York 10029, USA. [166]PharmaTherapeutics Clinical Research, Pfizer Worldwide Research and Development, Cambridge, Massachusetts 02139, USA. [167]Department of

Psychiatry and Psychotherapy, University of Gottingen, 37073 Gottingen, Germany. [168]Psychiatry and Psychotherapy Clinic, University of Erlangen, 91054 Erlangen, Germany. [169]Hunter New England Health Service, Newcastle NSW 2308, Australia. [170]School of Biomedical Sciences, University of Newcastle, Newcastle NSW 2308, Australia. [171]Division of Cancer Epidemiology and Genetics, National Cancer Institute, Bethesda, Maryland 20892, USA. [172]University of Iceland, Landspitali, National University Hospital, 101 Reykjavik, Iceland. [173]Department of Psychiatry and Drug Addiction, Tbilisi StateMedical University (TSMU), N33,0177Tbilisi,Georgia. [174]Research andDevelopment, BronxVeteransAffairsMedical Center, New York, New York 10468, USA. [175]Wellcome Trust Centre for Human Genetics, Oxford OX3 7BN, UK. [176]deCODE Genetics, 101 Reykjavik, Iceland. [177]Department of Clinical Neurology, Medical University of Vienna, 1090 Wien, Austria. [178]Lieber Institute for Brain Development, Baltimore, Maryland 21205, USA. [179]Department of Medical Genetics, University Medical Centre Utrecht, Universiteitsweg 100, 3584 CG, Utrecht, The Netherlands. [180]Berkshire Healthcare NHS Foundation Trust, Bracknell RG12 1BQ, UK. [181]Section of Psychiatry, University of Verona, 37134 Verona, Italy. [182]Department of Psychiatry, University of Oulu, P.O. Box 5000, 90014, Finland. [183]University Hospital of Oulu, P.O. Box 20, 90029 OYS, Finland. [184]Molecular and Cellular Therapeutics, Royal College of Surgeons in Ireland, Dublin 2, Ireland. [185]Health Research Board, Dublin 2, Ireland. [186]School of Psychiatry and Clinical Neurosciences, The University of Western Australia, Perth WA6009, Australia. [187]Computational Sciences CoE, Pfizer Worldwide Research and Development, Cambridge, Massachusetts 02139, USA. [188]Human Genetics, GenomeInstitute of Singapore, A*STAR, Singapore 138672. [189] [190]University College London, London WC1E 6BT, UK. [191]Department of Neuroscience, Icahn School of Medicine at Mount Sinai, NewYork, New York 10029, USA. [192]Institute of Neuroscience and Medicine (INM-1), Research Center Juelich, 52428 Juelich, Germany. [193]Department of Genetics, The HebrewUniversity of Jerusalem, 91905Jerusalem, Israel. [194]Neuroscience Discovery and Translational Area, Pharma Research and Early Development, F. Hoffman-La Roche, CH-4070 Basel, Switzerland. [195]Centre for Clinical Research in Neuropsychiatry, School of Psychiatry and Clinical Neurosciences, The University of Western Australia, Medical Research Foundation Building, Perth WA6000, Australia. [196]Virginia Institute for Psychiatric and Behavioral Genetics, Departments of Psychiatry and Human and Molecular Genetics, Virginia Commonwealth University, Richmond, Virginia 23298, USA. [197]The Feinstein Institute for Medical Research, Manhasset, New York 11030, USA. [198]The Hofstra NS-LIJ School of Medicine, Hempstead, New York 11549, USA. [199]The Zucker Hillside Hospital, Glen Oaks, New York 11004, USA. [200]Saw Swee Hock School of Public Health, National University of Singapore, Singapore 117597, Singapore. [201]Queensland Centre for Mental Health Research, University of Queensland, Brisbane 4076, Queensland, Australia. [202]Center for Human Genetic Research and Department of Psychiatry, Massachusetts General Hospital, Boston, Massachusetts 02114, USA. [203]Department of Child and Adoles-

cent Psychiatry, Erasmus University Medical Centre, Rotterdam 3000, The Netherlands. [204]Department of Complex Trait Genetics, Neuroscience Campus Amsterdam, VU University Medical Center Amsterdam, Amsterdam 1081, The Netherlands. [205]Department of Functional Genomics, Center for Neurogenomics and Cognitive Research, Neuroscience Campus Amsterdam, VU University, Amsterdam 1081, The Netherlands. [206]University of Aberdeen, Institute of Medical Sciences, Aberdeen AB25 2ZD, UK. [207]Departments of Psychiatry, Neurology, Neuroscience and Institute of Genetic Medicine, Johns Hopkins School of Medicine, Baltimore, Maryland 21205, USA. [208]Department of Clinical Medicine, University of Copenhagen, Copenhagen 2200, Denmark.

References

1. Yang J, Ferreira T, Morris AP, Medland SE, Madden PA, et al. (2012) Conditional and joint multiple-snp analysis of gwas summary statistics identifies additional variants influencing complex traits. Nature genetics 44: 369–375.

2. Zhou X, Carbonetto P, Stephens M (2013) Polygenic modeling with bayesian sparse linear mixed models. PLoS genetics 9: e1003264.

3. Franke A, McGovern DP, Barrett JC, Wang K, Radford-Smith GL, et al. (2010) Genome-wide meta-analysis increases to 71 the number of confirmed crohn's disease susceptibility loci. Nature genetics 42: 1118–1125.

4. of the Psychiatric Genomics Consortium SWG, et al. (2014) Biological insights from 108 schizophrenia-associated genetic loci. Nature 511: 421–427.

5. DerSimonian R, Laird N (1986) Meta-analysis in clinical trials. Controlled clinical trials 7: 177–188.
