# Supplementary figures and images for: An Empirical Bayes Mixture Model for Effect Size Distributions in Genome-Wide Association Studies
Source: PLoS Genet. 2015 Dec 29;11(12):e1005717. doi: 10.1371/journal.pgen.1005717 (PMC5456456; doi:10.1371/journal.pgen.1005717)

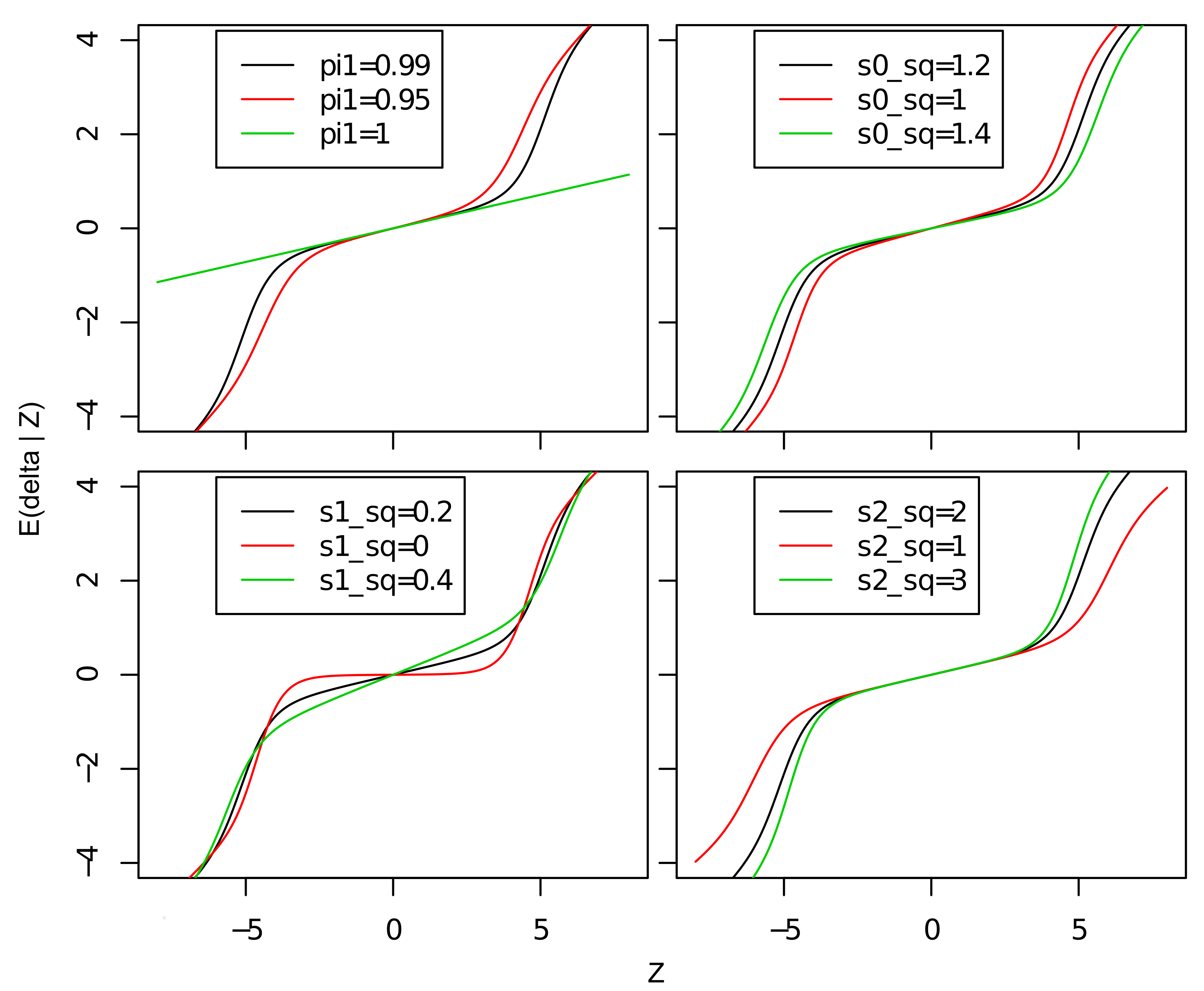

Supplement: S2 Fig — Expected posterior effect sizes for normal mixture model for different parameter values. Note, when σ11=0, the line through the origin is flat (has no positive slope). Black line denotes replication effect sizes for the same settings in each plot. (TIF) [file pgen.1005717.s003.tif]
